# HIV incidence and impact of interventions among female sex workers and their clients in the Middle East and north Africa: a modelling study

**DOI:** 10.1016/S2352-3018(22)00100-X

**Published:** 2022-06-28

**Authors:** Hiam Chemaitelly, Houssein H Ayoub, Ryosuke Omori, Shereen El Feki, Joumana G Hermez, Helen A Weiss, Laith J Abu-Raddad

**Affiliations:** a Infectious Disease Epidemiology Group, Weill Cornell Medicine-Qatar, Cornell University, Qatar Foundation-Education City, Doha, Qatar; b World Health Organization Collaborating Centre for Disease Epidemiology Analytics on HIV/AIDS, Sexually Transmitted Infections, and Viral Hepatitis, Weill Cornell Medicine-Qatar, Cornell University, Qatar Foundation-Education City, Doha, Qatar; c Department of Population Health Sciences, Weill Cornell Medicine, Cornell University, New York, NY, USA; d Department of Infectious Disease Epidemiology, Faculty of Epidemiology and Population Health, London School of Hygiene and Tropical Medicine, London, UK; e MRC International Statistics and Epidemiology Group, London School of Hygiene and Tropical Medicine, London, UK; f Mathematics Program, Department of Mathematics, Statistics, and Physics, College of Arts and Sciences, Qatar University, Doha, Qatar; g Department of Public Health, College of Health Sciences, QU Health, Qatar University, Doha, Qatar; h Division of Bioinformatics, Research Center for Zoonosis Control, Hokkaido University, Sapporo, Hokkaido, Japan; i Regional Support Team for the Middle East and North Africa, The Joint United Nations Programme on HIV/AIDS, Cairo, Egypt; j Department of Communicable Diseases Prevention and Control, World Health Organization Regional Office for the Eastern Mediterranean, Cairo, Egypt

## Abstract

**Background:**

The incidence of HIV infection among female sex workers and their clients in the Middle East and north Africa is not well known. We aimed to assess HIV incidence, the contribution of heterosexual sex work networks to these numbers, and the effect of interventions by use of mathematical modelling.

**Methods:**

In this modelling study, we developed a novel, individual-based model to simulate HIV epidemic dynamics in heterosexual sex work networks. We applied this model to 12 countries in the Middle East and north Africa that had sufficient data to estimate incidence in 2020 and the impact of interventions by 2030 (Algeria, Bahrain, Djibouti, Iran, Libya, Morocco, Pakistan, Somalia, South Sudan, Sudan, Tunisia, and Yemen). Model-input parameters were provided through a systematic review of HIV prevalence, sexual and injecting behaviours, and risk group size estimates of female sex workers and clients. Model output was number of incident HIV infections under different modelling scenarios for each country. Summary statistics were generated on these model output scenarios.

**Findings:**

Based on the output of our model, we estimated a total of 14 604 (95% uncertainty interval [UI] CI 7929–31 819) new HIV infections in the 12 countries in 2020 among female sex workers, clients, and spouses, which constituted 28·1% of 51 995 total new cases in all adults in these 12 countries combined. Model-estimated number of new infections in 2020 in the 12 countries combined was 3471 (95% UI 1295–10 308) in female sex workers, 6416 (3144–14 223) in clients, and 4717 (3490–7288) in client spouses. Contribution of incidence in heterosexual sex work networks to total incidence varied widely, ranging from 3·3% in Pakistan to 71·8% in South Sudan and 72·7% in Djibouti. Incidence in heterosexual sex work networks was distributed roughly equally among female sex workers, clients, and client spouses. Estimated incidence rates among female sex workers per 1000 person-years ranged from 0·4 (95% UI 0·0–7·1) in Yemen to 34·3 (17·2–59·6) in South Sudan. In countries where HIV acquisition through injecting drug use creates substantial exposure for female sex workers who inject drugs, estimated incidence rates per 1000 person-years ranged from 5·1 (95% UI 0·0–35·1) in Iran to 45·8 (0·0–428·6) in Pakistan. The model output predicted that any of the programmed interventions would substantially reduce incidence. Even when a subpopulation did not benefit directly from an intervention, it benefited indirectly through reduction in onward transmission, and indirect impact was often half as large as the direct impact.

**Interpretation:**

Substantial HIV incidence occurs in heterosexual sex work networks across the Middle East and north Africa with client spouses being heavily affected, in addition to female sex workers and clients. Rapid scaling-up of comprehensive treatment and prevention services for female sex workers is urgently needed.

**Funding:**

Qatar National Research Fund (a member of Qatar Foundation), the Biostatistics, Epidemiology, and Biomathematics Research Core at the Weill Cornell Medicine-Qatar, Qatar University-Marubeni, the UK Medical Research Council, and the UK Department for International Development.

## Introduction

To accelerate ending the public health threat of an HIV epidemic by 2030,^1^ UNAIDS formulated the UNAIDS 2016–2021 Strategy^2^ and the Global AIDS Strategy 2021–2026^3^ as calls for scaling up HIV response among people living with HIV to achieve 90% coverage for HIV testing, treatment, and sustained viral suppression by 2020^2^ and 95% coverage by 2030.^2^, ^3^ The strategy emphasised enhancing access to combination prevention interventions among key populations as a cornerstone to this goal.^2^ Targets were set to reduce the number of people newly acquiring HIV and of AIDS-related deaths to fewer than 500 000 per year by 2020 and fewer than 200 000 by 2030.^2^, ^3^

Despite progress, the global community has not met the 2020 targets, with 1**·**5 million new HIV infections and 680 000 AIDS-related deaths estimated in 2020.^4^ Over half of newly acquired infections occurred among key populations and their sexual partners,^5^ indicating persistent gaps in reaching populations most at risk.^6^

**Research in context**

**Evidence before this study**

The burden of HIV is growing in the Middle East and north Africa. Despite evidence for emerging epidemics among female sex workers in the Middle East and north Africa, HIV incidence among them and their clients is unknown. We searched PubMed and Embase for all publicationsfrom database inception to Sept 9, 2021, with no language restriction and using broad terms for sex work, HIV, and the Middle East and north Africa (appendix pp 3, 4). We identified no regional estimates for HIV incidence among female sex workers and their clients.

**Added value of this study**

We used a novel individual-based mathematical model to describe HIV transmission dynamics in heterosexual sex work networks in the Middle East and north Africa to estimate HIV incidence and the potential impact of interventions among female sex workers, clients, and client spouses. Although incidence of HIV is more likely to be detected among female sex workers, it constitutes only a third of the incidence in heterosexual sex work networks—the other two-thirds are split among clients and their spouses, who rarely access any HIV programmes. Our results suggest that clients and their spouses could benefit greatly from expanding coverage of interventions, even if these interventions are delivered only to female sex workers. These estimates inform HIV programming and monitoring of progress toward achieving UNAIDS targets.

**Implications of all the available evidence**

With the emergence of HIV epidemics in female sex workers in the Middle East and north Africa, HIV incidence in heterosexual sex work networks is likely to increase. Scale-up of interventions among female sex workers should be a priority, and such interventions will have a substantial impact on reducing infection burden among female sex workers, their clients, client spouses, and other women in the general population. Strengthening non-governmental entities that work with female sex workers to deliver services and programmes might help, as it has in several countries. Surveillance systems for HIV need to be enhanced among female sex workers through regular, national, integrated biobehavioural surveillance surveys to monitor the HIV epidemic and progress toward global targets and to enhance our understanding of HIV epidemiology in heterosexual sex work networks.

The Middle East and north Africa, a region including approximately 10% of the world's population, continues to lag behind in HIV prevention and treatment.^6^ Antiretroviral therapy (ART) coverage, as defined by UNAIDS, is only 43% in the Middle East and north Africa, the lowest across all world regions.^6^ HIV incidence seems to have increased since 2010.^6^ HIV epidemics have emerged over the past 20 years in female sex workers,^7^ men who have sex with men,^8^ and people who inject drugs.^9^ Yet, HIV surveillance remains limited in scale and scope,^7^, ^8^, ^9^ with scarce data on incidence among marginalised and hard-to-reach populations.^7^, ^8^, ^9^ Heterosexual sex work networks might be driving a large proportion of HIV incidence in the Middle East and north Africa because they constitute a larger population^7^ than people who inject drugs^9^ or men who have sex with men.^8^ However, HIV incidence among female sex workers and their clients remain unknown.^7^ This evidence gap, along with the punitive laws and widespread stigma and discrimination affecting female sex workers, continue to hamper HIV programming and monitoring of progress toward UNAIDS targets.

To address this evidence gap, we developed a novel individual-based mathematical model to simulate HIV transmission dynamics in heterosexual sex work networks and applied it to each country in the Middle East and north Africa to estimate current HIV incidence and incidence rate among female sex workers, their clients, and client stable sexual partners or spouses; relative contribution of heterosexual sexual intercourse versus injecting drug use to incidence among female sex workers; contribution of heterosexual sex work networks to incidence in the total adult population; and impact of various targets for interventions on incidence in heterosexual sex work networks. Although this model is novel in its structure and in its focus on HIV dynamics in heterosexual sex work networks in the Middle East and north Africa, it can also be adapted and applied to other countries or regions and for other sexually transmitted infections.

## Methods

### Overview of mathematical model

**Figure 1:**
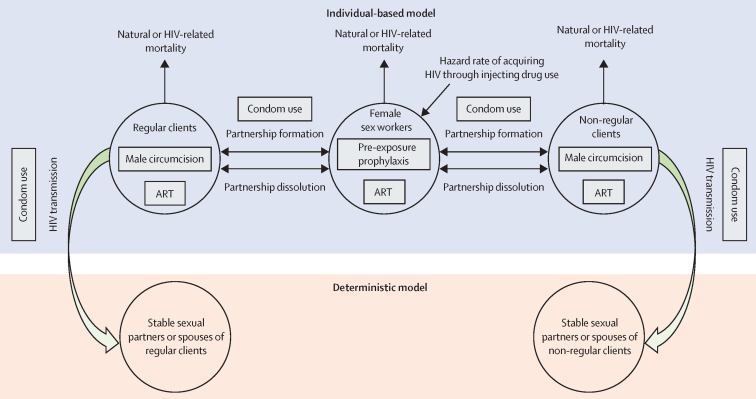
Basic structure of the model describing HIV transmission dynamics in female sex workers, clients, and client spouses ART=antiretroviral therapy.

We developed an individual-based Monte Carlo simulation model to simulate sexual networks of female sex workers and clients and HIV transmission dynamics in these networks, and to estimate current and future HIV incidence, factoring in current intervention coverage and potential future scale-up (figure 1). Model structure was informed by earlier individual-based models for sexually transmitted infections.^10^, ^11^ The model simulates cohorts of female sex workers and clients (regular and non-regular [ie, single-visit]) in each country over time as they engage in sexual behaviours (and injecting for female sex workers) and acquire or transmit HIV.

Mathematical modelling methods, including the heterosexual sex work network, HIV sexual transmission in female sex worker–client partnerships, HIV transmission through drug injection, HIV natural history, and outcome measures are described in the appendix (pp 5–12). HIV estimation methods for HIV sexual transmission from clients to their spouses are shown in the appendix (pp 16–17). The model was coded and the fitting and all analyses were done in MATLAB R2019a (Massachusetts, USA). Simulations were done using the Red Cloud infrastructure of Cornell University (appendix pp 18–19).

### Data sources and model parameters

The primary data source for this modelling study was a recently completed systematic review^7^ of HIV prevalence and sexual and injecting behaviours among female sex workers and clients in the Middle East and north Africa and size estimates of these populations. The review identified 485 HIV prevalence measures on 287 719 female sex workers and 69 measures on 29 531 clients and proxy populations, along with detailed sexual and injecting behaviour data and more than 300 population-size estimates in these populations.

We included countries if they had sufficient input data to simulate the HIV epidemic in the heterosexual sex work network, and if HIV prevalence among female sex workers was at least 0·5%. Otherwise, it was not feasible to conduct the simulations. 12 countries were included (Algeria, Bahrain, Djibouti, Iran, Libya, Morocco, Pakistan, Somalia, South Sudan, Sudan, Tunisia, and Yemen; appendix p 23). Injecting drug use among female sex workers was modelled in countries in which evidence suggested a substantial role for injecting drug use in the HIV epidemic (Bahrain, Iran, Libya, and Pakistan).^7^

In this model, sexual behaviour data were used as input to simulate the structure of the sexual network. Model simulations were generated to fit HIV prevalence among female sex workers and among female sex workers who inject drugs, thereby determining the distribution of HIV infection in the simulated populations. Risk group size estimates were used to set the size of populations for each country. We ran the model to provide estimates for the number of new infections in 2020. A listing of the model parameters can be found in the appendix (pp 13–15). Data sources and model parameters are detailed in the appendix (pp 5–12).

### Model simulations, model fitting, and intervention impact

We established the model-generated sexual network with a burn-in of 50 years to ensure equilibrium of network structure before HIV introduction. Subsequently, HIV infection was seeded and the model was run for an additional burn-in of 300 years to ensure epidemic equilibrium in each country by 2020. Model predictions for each country were generated assuming endemic equilibrium and were based on the mean and 95% uncertainty intervals (UIs) of distributions of outcome measures generated by 500 simulation runs (appendix pp 9–10). At this number of runs, the mean and distribution of outcome measures varied minimally with an increase in the number of simulations. 95% UIs were generated directly from these distributions of simulation runs after excluding runs with HIV stochastic extinction. For computational efficiency, after experimenting with different cohort sizes, simulations were performed using a cohort of 600 female sex workers and 6000 clients (one-third of whom were regular and two-thirds were non-regular or one-time clients), as informed by the Middle East and north Africa data.^7^ Outcome measures were subsequently scaled up to reflect the actual population sizes in each country.^7^

We did model fitting to HIV prevalence data among female sex workers and HIV prevalence among female sex workers who inject drugs to estimate the overall sexual partnership formation rate and the baseline hazard rate of acquiring HIV through injecting drug use in each included country. We implemented nonlinear least-square fitting using the Nelder-Mead simplex algorithm^12^ iteratively to generate a set of 50 best model fits. A best model fit was defined as a relative error of less than 5% between model predictions and empirical data. The final best model fit was the most probable value for the sexual partnership rate and injecting hazard rate among the 50 best model fits, rather than means or medians as these resulted in inferior fits. Examples of the model fitting for Morocco (a country with no substantial HIV transmission through injecting drug use among female sex workers) and Iran (a country with significant HIV transmission through injecting drug use among female sex workers) can be found in the appendix (pp 25–26).

We assessed the impact of expanding HIV interventions among female sex workers on HIV incidence arising in heterosexual sex work networks by estimating the number of infections that would be averted over a 10-year period after implementation of the interventions, and the proportional decrease in incidence during this time. These interventions included ART among female sex workers and their injecting partners, condom use in the partnership, voluntary medical male circumcision by the client, and the use of pre-exposure prophylaxis (PrEP) by the female sex workers. Current coverage of these interventions for female sex workers and clients was based on data for each country. Model simulations and fitting are detailed in the appendix (pp 9–11).

### Role of the funding source

The funder of the study had no role in study design, data collection, data analysis, data interpretation, or writing of the article. The corresponding author had full access to all the data in the study and had the final responsibility for the decision to submit for publication.

## Results

We estimated a total of 14 604 (95% UI 7929–31 819) new HIV infections in the 12 countries in 2020 among female sex workers, clients, and spouses, which constituted 28·1% of 51 995 total new cases in all adults in these 12 countries combined,^13^ and 25·1% of 58 189 estimated cases in adults in all 23 countries of the Middle East and north Africa (appendix p 27). Of these 14 604 new infections, 3471 (95% UI 1295–10 308) were in female sex workers, 6416 (3144–14 223) were in clients, and 4717 (3490–7288) were in client spouses (Table 1, Table 2).

**Table 1:** HIV epidemiological measures for female sex workers, clients, and client spouses in countries in the Middle East and north Africa with no significant HIV transmission through injecting drug use among female sex workers, 2020

				**Algeria**	**Djibouti**	**Morocco**	**Somalia**	**South Sudan**	**Sudan**	**Tunisia**	**Yemen**
**Data used in the model and obtained from sources external to the model**											
Population											
	Female sex workers (n)			65 969	4481	72 000	36 174	110 968	212 500	25 500	58 934
	Female sex workers (proportion; %)*			0·6%	1·7%	0·8%	1·0%	4·1%	2·0%	0·9%	1·6%
Clients of female sex workers (n)				659 690	44 810	720 000	361 740	1 109 680	2 125 000	255 000	589 340
HIV prevalence among all female sex workers (empirical data; %)				4·9%	9·3%	2·2%	4·5%	37·9%	1·5%	1·2%	0·8%
HIV incidence in the total adult population per year as estimated by UNAIDS† (n)				2000	<100	<1000	<500	16 000	2900	<1000	1000
Current HIV intervention coverage (%)											
	Condom use (empirical data)			65·3%	59·6%	52·3%	31·5%	72·4%	26·0%	58·3%	46·0%
	Male circumcision (empirical data)			97·9%	96·5%	99·9%	93·5%	23·6%	90·7%	99·8%	99·0%
		ART (empirical data)									
			Female sex workers	32·0%	30·0%	57·0%	28·0%	9·4%	15·0%	31·0%	21·0%
			Clients or people living with HIV	32·0%	30·0%	57·0%	28·0%	16·0%	15·0%	31·0%	21·0%
		PrEP (empirical data)									
			Female sex workers	0·0%	0·0%	0·0%	0·0%	0·0%	0·0%	0·0%	0·0%
			Clients	0·0%	0·0%	0·0%	0·0%	0·0%	0·0%	0·0%	0·0%
**Model estimates for 2020**											
HIV prevalence (%)											
	All female sex workers			4·9% (0·8–12·8%)	9·2% (3·3–16·0%)	2·2% (0·5–8·0%)	4·6% (0·8–13·1%)	38·2% (32·2–43·5%)	1·5% (0·3–9·7%)	1·4% (0·2–8·3%)	0·7% (0·2–6·0%)
	Clients of female sex workers			1·3% (0·2–3·3%)	2·4% (0·8–4·3%)	0·5% (0·1–1·9%)	1·1% (0·2–3·0%)	16·9% (14·0–19·2%)	0·3% (0·07–2·3%)	0·4% (0·07–2·1%)	0·2% (0·1–1·7%)
	Client spouses			0·4% (0·1–1·1%)	0·8% (0·3–1·4%)	0·2% (0·03–0·6%)	0·4% (0·1–1·0%)	5·6% (4·7–6·4%)	0·1% (0·02–0·8%)	0·1% (0·02–0·7%)	0·06% (0·0–0·6%)
HIV incidence in heterosexual sex work networks per year (n)											
	All female sex workers			179 (0–770)	21 (0–60)	83 (0–600)	93 (0–422)	2345 (1295–3884)	163 (0–1771)	21 (0–170)	26 (0–393)
	Clients of female sex workers			234 (0–770)	29 (0–67)	100 (0–600)	113 (0–422)	5167 (3144–7398)	213 (0–2125)	25 (0–213)	30 (0–393)
	Client spouses			173 (31–431)	22 (7–39)	61 (11–217)	84 (15–266)	3978 (3330–4484)	166 (32–1082)	18 (4–108)	26 (10–235)
HIV incidence rate‡ (per 1000 person–years)											
	All female sex workers			2·9 (0·0–13·2)	5·1 (0·0–14·8)	1·2 (0·0–8·7)	2·8 (0·0–12·5)	34·3 (17·2–59·6)	0·8 (0·0–8·8)	0·9 (0·0–7·3)	0·4 (0·0–7·1)
	Clients of female sex workers			0·2 (0·0–0·6)	0·3 (0·0–0·8)	0·07 (0·0–0·4)	0·2 (0·0–0·6)	2·5 (1·5–3·6)	0·05 (0·0–0·5)	0·05 (0·0–0·4)	0·03 (0·0–0·3)
	Client spouses			0·5 (0·08–1·2)	0·9 (0·3–1·6)	0·2 (0·03–0·6)	0·4 (0·07–1·1)	6·7 (5·6–7·7)	0·1 (0·03–0·9)	0·1 (0·02–0·8)	0·07 (0·03–0·7)
Contribution to total HIV incidence in the population (%)											
	All female sex workers			9·0% (0·0–38·5%)	21·2% (0·0–60·6%)	8·3% (0·0–60·1%)	18·6% (0·0–84·6%)	14·7% (8·1–24·3%)	5·6% (0·0–61·1%)	2·1% (0·0–17·0%)	2·6% (0·0–39·3%)
	Clients of female sex workers			11·7% (0·0–38·5%)	29·3% (0·0–67·7%)	10·0% (0·0–60·1%)	22·6% (0·0–84·6%)	32·3% (19·7–46·2%)	7·3% (0·0–73·3%)	2·5% (0·0–21·3%)	3·0% (0·0–39·3%)
	Client spouses			8·7% (1·6–21·6%)	22·2% (7·1–39·4%)	6·1% (1·1–21·7%)	16·8% (3·0–53·3%)	24·9% (20·8–28·0%)	5·7% (1·1–37·3%)	1·8% (0·4–10·8%)	2·6% (1·0–23·5%)
	Heterosexual sex work networks			29·3% (1·6–98·6%)	72·7% (7·1–100%)	24·4% (1·1–100%)	58·1% (3·0–100%)	71·8% (48·6–98·5%)	18·7% (1·1–100%)	6·4% (0·4–49·1%)	8·2% (1·0–100%)

Data are n (95% UI), or % (95% UI) unless otherwise specified. Numbers are rounded to the first decimal unless the number was <0·1%. The table includes measures based on empirical data for model input, as well as measures estimated using the model. ART=antiretroviral therapy. PrEP=pre-exposure prophylaxis. UI=uncertainty interval.

* Proportion of female sex workers out of total reproductive-age women aged 15–49 years.

† Estimates for the number of new infections occurring in the population per year were provided by UNAIDS.^26^ Assumed to be 99 where incidence is reported as <100, 499 where incidence is reported as <500, and 999 where incidence is reported as <1000.

‡ Numbers of new HIV infections per susceptible person per 1000 person-years.

**Table 2:** HIV epidemiological measures among female sex workers, clients, and client spouses in countries in the Middle East and north Africa with significant HIV transmission through injecting drug use among female sex workers, 2020

			**Bahrain**	**Iran**	**Libya**	**Pakistan**
**Data used in the model and obtained from sources external to the model**						
Population						
	Female sex workers (n)		2143	91 500	11 459	228 800
	Female sex workers (proportion; %)*		0·6%	1·4%	0·6%	0·4%
	Clients of female sex workers (n)		21 430	915 000	114 590	2 288 000
	Proportion of female sex workers who inject drugs (%)		3·9%	13·6%	2·9%	2·0%
HIV prevalence (empirical, %)						
	All female sex workers		0·8%	3·3%	4·9%	2·3%
	Female sex workers who inject drugs		21·0%	9·9%	44·0%	38·4%
HIV incidence in the total adult population per year as estimated by UNAIDS† (n)			Unknown	4000	<500	23 000
Current HIV interventions' coverage (%)						
	Condom use (empirical data)		44·0%	57·1%	80·0%	50·5%
	Male circumcision (empirical data)		81·2%	99·7%	96·6%	96·4%
	ART (empirical data)					
		Female sex workers	45·0%	20·0%	44·0%	8·0%
		Clients or people living with HIV	45·0%	20·0%	44·0%	8·0%
	PrEP (empirical data)					
	Female sex workers		0·0%	0·0%	0·0%	0·0%
	Clients		0·0%	0·0%	0·0%	0·0%
**Model estimates for 2020**						
HIV prevalence (%)						
	All female sex workers		0·9% (0·3–1·8%)	3·3% (1·3–6·3%)	4·6% (1·8–8·3%)	2·4% (0·7–5·0%)
	Female sex workers who inject drugs		20·2% (8·0–37·0%)	9·9% (3·4–17·8%)	44·8% (21·1–68·8%)	37·8% (11·1–66·7%)
	Clients of female sex workers		0·03% (0·0–0·08%)	0·3% (0·1–0·6%)	0·5% (0·2–1·0%)	0·2% (0·05–0·6%)
	Client spouses		0·01% (0·0–0·03%)	0·1% (0·03–0·2%)	0·2% (0·07–0·3%)	0·08% (0·02–0·2%)
HIV incidence in heterosexual sex work networks per year (n)						
	All female sex workers		1 (0–7)	172 (0–610)	28 (0–96)	339 (0–1525)
	Female sex workers who inject drugs		1 (0–7)	55 (0–305)	7 (0–38)	110 (0–763)
	Clients of female sex workers		<1 (0–4)	171 (0–610)	33 (0–96)	301 (0–1525)
	Client spouses		<1 (0–1)	64 (20–127)	11 (5–20)	114 (25–278)
HIV incidence rate‡ (per 1000 person-years)						
	All female sex workers		0·5 (0·0–3·4)	2·0 (0·0–7·1)	2·6 (0·0–8·8)	1·5 (0·0–6·9)
	Female sex workers who inject drugs		15·2 (0·0–117·6)	5·1 (0·0–35·1)	43·4 (0·0–300·0)	45·8 (0·0–428·6)
	Clients of female sex workers		0·02 (0·0–0·2)	0·2 (0·0–0·7)	0·3 (0·0–0·8)	0·1 (0·0–0·7)
	Client spouses		0·01 (0·0–0·03)	0·1 (0·04–0·3)	0·2 (0·07–0·3)	0·09 (0·02–0·2)
Contribution to HIV incidence in female sex workers (%)‡						
Sexual transmission			14·5%	68·0%	75·0%	67·6%
Injecting drug use			85·5%	32·0%	25·0%	32·4%
Contribution to total HIV incidence in the population (%)						
	All female sex workers§		..	4·3% (0·0–15·3%)	5·6% (0·0–19·2%)	1·5% (0·0–6·6%)
	Injecting drug use in female sex workers		..	1·4% (0·0–7·6%)	1·4% (0·0–7·6%)	0·5% (0·0–3·3%)
	Clients of female sex workers		..	4·3% (0·0–15·3%)	6·6% (0·0–19·2%)	1·3% (0·0–6·6%)
	Client spouses		..	1·6% (0·5–3·2%)	2·2% (1·0–4·0%)	0·5% (0·1–1·2%)
	Heterosexual sex work networks		..	10·2% (0·5–33·7%)	14·4% (1·0–42·5%)	3·3% (0·1–14·5%)

Data are n (95% UI) or % (95% UI) unless otherwise specified. Numbers are rounded to the first decimal unless the number was <0·1%. The table includes measures based on empirical data for model input, as well as measures estimated using the model. ART=antiretroviral therapy. PrEP=pre-exposure prophylaxis. UI=uncertainty interval.

* Proportion of female sex workers out of total reproductive-age women aged 15–49 years.

† Estimates for the number of new infections occurring in the population per year were provided by UNAIDS.^26^ Assumed to be 499 where incidence is reported as <500.

‡ Numbers of new HIV infections per susceptible person per 1000 person-years.

§ Including female sex workers who inject drugs.

For countries in which HIV acquisition through injecting drug use among female sex workers is negligible, estimated numbers of new infections among female sex workers in 2020 ranged from 21 in Djibouti to 2345 in South Sudan (table 1). Estimated numbers of new infections in clients ranged from 25 in Tunisia to 5167 in South Sudan, whereas estimated new infections among client spouses ranged from 18 in Tunisia to 3978 in South Sudan.

Although the estimated number of incident infections by country varied widely with the size of heterosexual sex work networks, the total incidence in heterosexual sex work networks in each country was distributed roughly equally among female sex workers, clients, and client spouses (table 1). The only exception was South Sudan (the only country in this region with low male circumcision coverage),^14^ where incidence in clients and their spouses was twice that in female sex workers (table 1). In all countries other than South Sudan, HIV prevalence in clients was approximately a quarter of that in female sex workers. Incidence in heterosexual sex work networks as a proportion of the total incidence in the population ranged from 6·4% in Tunisia to 71·8% in South Sudan and 72·7% in Djibouti. Incidence rates among female sex workers ranged from 0·4 (95% UI 0·0–7·1) per 1000 person-years in Yemen to 34·3 (17·2–59·6) per 1000 person-years in South Sudan.

In countries where HIV acquisition through injecting drug use creates substantial exposure for female sex workers, estimated numbers of new infections among female sex workers in 2020 ranged from one in Bahrain to 339 in Pakistan (table 2). Estimated new infections in clients and their spouses ranged from less than one in Bahrain to 301 in clients and 114 in clients’ spouses in Pakistan. Incidence among female sex workers out of total incidence in heterosexual sex work networks was higher in countries with high drug injection transmission (table 2) than in countries with low drug injection transmission (table 1), as many female sex workers were infected through drug injection in addition to those being infected through sex. However, sexual transmission contributed most HIV incidence among female sex workers (eg, 67·6% in Pakistan, 68·0% in Iran, and 75·0% in Libya; table 2).

Incidence of new HIV infection among clients and their spouses was a smaller proportion of the total incidence in the heterosexual sex work networks in these countries because of the role of injecting. In these countries, HIV prevalence in clients was about 10% of that in female sex workers (table 2). The contribution of heterosexual sex work networks to total incidence in the population was also low in these countries compared with those countries with low drug injection transmission, ranging from 3·3% in Pakistan to 14·4% in Libya. Incidence rate per 1000 person-years among all female sex workers (including those who inject drugs) ranged from 0·5 (95% UI 0·0–3·4) in Bahrain to 2·6 (0·0–8·8) in Libya. However, female sex workers who inject drugs had disproportionately high incidence rates per 1000 person-years, ranging from 5·1 (95% UI 0·0–35·1) in Iran to 45·8 (0·0–428·6) in Pakistan.

**Figure 2:**
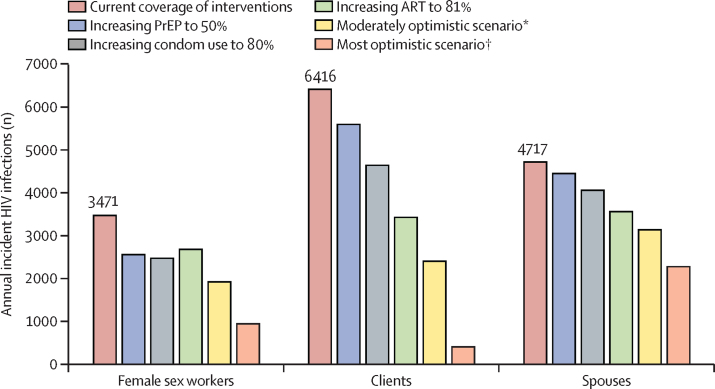
Estimated effect of expanding coverage of HIV prevention and treatment interventions among female sex workers on HIV incidence in the Middle East and north Africa PrEP=pre-exposure prophylaxis. ART=antiretroviral therapy. *Moderately optimistic scenario that includes expanding PrEP to 25%, condom use to 50%, ART to 50% (assuming efficacy of 96%, that is optimal adherence), and voluntary male circumcision to 50% (in South Sudan). †Most optimistic scenario that includes expanding PrEP to 50%, condom use to 80%, ART to 81% (assuming efficacy of 96%, that is optimal adherence), and voluntary male circumcision to 80% (in South Sudan).

Our models show that all considered interventions, whether individually or in combination, would substantially reduce incidence among female sex workers, clients, and client spouses (figure 2 and appendix pp 28–30). However, the interventions would affect the three subpopulations differently. Increasing ART coverage and improving adherence to treatment among female sex workers would result in major reductions in incidence, with clients benefiting directly from viral suppression in HIV-positive female sex workers. Female sex workers and client spouses would benefit only indirectly through a reduction in the pool of HIV-positive clients. However, the predicted number of averted infections among female sex workers and spouses is also substantial, and as much as half of that seen in clients in countries where HIV transmission through injecting drug use is negligible (appendix pp 28–29). In countries where HIV transmission through injecting drug use is a substantial mode of HIV exposure, female sex workers would also benefit directly from increased ART coverage and adherence, as this would increase viral suppression among their injecting partners (appendix p 30).

Our model predicted that increased condom use would considerably reduce incidence for both female sex workers and clients, as both benefit directly from this intervention (figure 2 and appendix pp 28–30). Although client spouses would not benefit directly from this intervention, the estimated number of averted infections among them was about half of that among clients (appendix pp 28–30) because of the reduction in the number of HIV-positive clients.

Expanding coverage of PrEP among female sex workers, which remains very scarce in the Middle East and north Africa,^5^ would reduce incidence considerably, with female sex workers benefiting most from the directly diminished risk of HIV acquisition (figure 2 and appendix pp 28–30). Clients would benefit indirectly by reducing the pool of HIV-positive female sex workers, but the estimated number of averted infections among clients was substantial, and as many as half of that in female sex workers. Client spouses would also have significantly reduced incidence through this already indirect benefit to clients. The numbers of predicted averted infections among spouses were around half of those among clients (appendix pp 28–29).

The model suggests that expansion of voluntary medical male circumcision coverage in South Sudan, the only country in the Middle East and north Africa where this intervention would probably provide a benefit, would lead to major reductions in HIV incidence among clients, spouses, and female sex workers (appendix pp 28–29). The predicted number of averted infections was particularly high for clients and their spouses.

Packages of combined interventions would reduce incidence considerably. A moderately optimistic combination of interventions (PrEP to 25%, condom use to 50%, ART to 50%, and voluntary male circumcision to 50% in South Sudan) would lead to a major reduction in incidence among female sex workers and clients (and half this reduction in client spouses), the most optimistic scenario (PrEP to 50%, condom use to 80%, ART to 81%, and voluntary male circumcision to 80% in South Sudan) would eliminate most new infections among female sex workers and clients, and half as many among spouses (table 3, figure 2, and appendix pp 28–30).

**Table 3:** Select modelled HIV prevention intervention packages to control HIV incidence in female sex workers and clients in the Middle East and north Africa

		**Coverage level scenario**
Expanding ART coverage in female sex workers and their injecting partners assuming real-world ART effectiveness in achieving viral suppression of 57% (real-world adherence to ART)^15^		Increase to 25%; increase to 50%; increase to 81% (global target)^7^
Expanding ART coverage in female sex workers and their injecting partners assuming ART efficacy in preventing HIV transmission to partners of 96% (optimal adherence to ART)^16^		Increase to 25%; increase to 50%; increase to 81% (global target)^7^
Increasing condom use coverage		Increase to 50%; increase to 80%
Expanding VMMC coverage in clients (only South Sudan)^14^		Increase to 50%; increase to 80%
Expanding PrEP coverage in female sex workers		Increase to 25%; increase to 50%
Moderately optimistic scenario		
Expanding ART coverage in female sex workers and their injecting partners assuming ART efficacy in preventing HIV transmission to partners of 96%; increasing condom use coverage; expanding VMMC coverage in clients (only South Sudan); expanding PrEP coverage in female sex workers		Expanding ART coverage to 50% with efficacy in preventing HIV transmission to partners of 96%, increasing condom use to 50%, and increasing PrEP to 25%. Baseline coverage was used whenever it was higher than that set in the investigated scenario. For South Sudan only, this package also included increasing VMMC to 50%
Most optimistic scenario		
	Expanding ART coverage in female sex workers and their injecting partners assuming ART efficacy in preventing HIV transmission to partners of 96%; increasing condom use coverage; expanding VMMC in clients (only South Sudan); expanding PrEP coverage in female sex workers	Expanding interventions to the highest modelled coverage levels, including expanding ART coverage to 81% with efficacy of 96%, increasing condom use to 80%, and increasing PrEP to 50%; for South Sudan only, this package also included increasing VMMC to 80%

ART=antiretroviral therapy. PrEP=pre-exposure prophylaxis. VMMC=voluntary medical male circumcision. Baseline coverage was used whenever it was higher than that set in the investigated scenario

## Discussion

HIV transmission in heterosexual sex work networks is a major source of incident cases in the Middle East and north Africa and contributes at least 25% of the annual number of all HIV infections in the 12 countries analysed. The contribution of heterosexual sex work networks to incidence varied among countries from 3% in Pakistan to over 70% in South Sudan and Djibouti. This variation reflected large differences in epidemic phase (recent or established epidemic), HIV prevalence among female sex workers, and HIV prevalence among other key populations. Even in countries where HIV prevalence among female sex workers is relatively low, substantial incidence occurs in heterosexual sex work networks due to their large size compared with networks of men who have sex with men and people who inject drugs. For example, HIV prevalence among female sex workers in Morocco is only 2%, but heterosexual sex work networks represent 24% of all incident cases in this country.

HIV incidence is more likely to be detected among female sex workers than among clients and their spouses because of limited HIV testing and prevention programming,^7^, ^17^, ^18^ and our findings suggest that incidence among female sex workers is estimated to constitute less than a third of the actual incidence that occurs in heterosexual sex work networks. The other two-thirds are split among clients and their spouses, who rarely access HIV response programming. A third of HIV incidence in heterosexual sex work networks occurs among spouses of clients, although they do not engage in sexual risk behaviour and do not normally benefit from HIV prevention interventions, but are exposed to infection by their spouses. This finding and vulnerability is consistent with evidence in the Middle East and north Africa that for almost all infections among women, the source of the infection is an HIV-positive spouse.^17^, ^19^, ^20^, ^21^, ^22^, ^23^

Against a background of expanding epidemics in heterosexual sex work networks, our results suggest that interventions can significantly reduce incidence and prevent expansion of epidemics. However, coverage of these interventions is low, and achieving the UNAIDS elimination target will require scale-up not only of single interventions, but of combinations.

Different interventions are variously beneficial to female sex workers, clients, or client spouses. Nonetheless, even when a subpopulation does not benefit directly from an intervention, it still benefits indirectly by reducing the pool of infected persons in the heterosexual sex work network. Indirect effects on onward transmission were large and often about half as large as the direct effects. In client spouses, for example, predicted reduction in incidence was often as large as half the reduction seen in clients or female sex workers, despite none of the interventions targeting them directly.

Despite substantial incidence arising in heterosexual sex work networks, the HIV response in the Middle East and north Africa is small in scope and scale.^18^ Our systematic review of HIV among female sex workers showed that only 18% of female sex workers report ever being tested for HIV,^7^ lower than that found in other regions^24^ and far below the 90% target of the UNAIDS strategy for 2021.^2^ ART coverage among people living with HIV in the Middle East and north Africa is the lowest of all world regions,^5^, ^6^ and far behind the WHO regional target of 50% coverage by 2015.^25^ We could not identify any data on viral suppression in female sex workers affected by HIV in the Middle East and north Africa.^5^, ^6^ The situation might have worsened with the COVID-19 pandemic due to interruptions in the provision of prevention and treatment services.^26^ The results also highlight an additional vulnerability for female sex workers who inject drugs, in whom as much as a third of HIV incidence was due to drug injection in countries such as Iran and Pakistan. Gender-sensitive harm reduction services for female sex workers who inject drugs need to be available wherever a substantial proportion of female sex workers inject drugs.

Reaching female sex workers and their clients in the Middle East and north Africa continues to be a challenge given punitive laws^18^, ^27^, ^28^ and stigma^29^, ^30^, ^31^ associated with sex work. These punitive laws continue to be a barrier to progress on HIV in the region. Diverse types (eg, facility-based, home-based, and street-based female sex workers) and increased movement of female sex workers^28^, ^32^, ^33^ are additional hurdles. Programmes and services, where they exist, are mainly under control of non-governmental organisations, which are often inadequately resourced or under legal restrictions preventing provision of comprehensive intervention packages to female sex workers.^17^, ^18^ Intervention usage strategies such as condom use in affected areas need to be readdressed, with reference to the specific epidemiology and potential impact of the interventions as described in our findings.

This study has limitations. Estimates were generated through mathematical modelling, and therefore should be seen as an approximation of reality. Data availability and quality varied across countries, possibly affecting the generalisability of estimates. In the absence of country-level trend data,^7^ estimates were generated assuming endemic equilibrium, which might not have had an appreciable effect on estimated epidemiological measures such as incidence, but could underestimate the impact of interventions if HIV prevalence is increasing, as suggested for the Middle East and north Africa region.^7^ For computational feasibility and efficiency, we did simulations with subcohorts of female sex workers and clients that are representative of the full cohorts of female sex workers and clients. However, sensitivity analyses revealed a limited effect for stochastic fade-out on simulated model outcomes (appendix p 24). Both point estimates and uncertainty ranges were obtained directly from model simulations and might not have factored in other sources of uncertainty that exist in real world sexual networks. These limitations are discussed in detail in the appendix (pp 20–22).

Heterosexual sex work networks contribute at least a quarter of annual HIV infections in the Middle East and north Africa. With the nascency of HIV epidemics among female sex workers, and evidence suggesting a trend of increasing HIV prevalence,^7^ HIV incidence in heterosexual sex work networks is likely to grow. Scale-up of interventions among female sex workers should be a priority, and this study forecasts a substantial effect for these interventions in controlling the epidemic. However, the region is still far from achieving UNAIDS targets, and the COVID-19 pandemic might have worsened the situations.^2^, ^6^ ART coverage must be scaled up rapidly among female sex workers, along with programmes that improve retention in the treatment cascade and their access to comprehensive prevention services. Strengthening the role of non-governmental entities working with female sex workers to lead the delivery of services and programmes, supported by Governments, could prove successful, as they have in Morocco.^7^, ^18^ Expansion of surveillance systems, including regular national integrated biobehavioural surveillance surveys, is crucial to monitor the epidemic and to track progress toward UNAIDS goals. This surveillance work needs also to include mapping and size estimation studies to delineate the diverse types of sex work, and to ensure evidence-informed response with adequate coverage of interventions.

## Data sharing

The input data used for this modelling study are based on a published systematic review of HIV prevalence and sexual and injecting behaviours among female sex workers and clients in the Middle East and north Africa, and size estimates of these populations.^7^ The source code of the study's mathematical model can be accessed at https://github.com/HousseinAyoub/Individual-based-model-for-HIV-among-FSWs-and-their-clients.git.

## Declaration of interests

We declare no competing interests.
