# Supplementary material for: HIV incidence and impact of interventions among female sex workers and their clients in the Middle East and north Africa: a modelling study
Source: Lancet HIV. 2022 Jun 28;9(7):e496–505. doi: 10.1016/S2352-3018(22)00100-X (PMC9253890; doi:10.1016/S2352-3018(22)00100-X)
Supplement: Supplementary appendix [file mmc1.pdf]

THE LANCET
HIV

Supplementary appendix

This appendix formed part of the original submission and has been peer reviewed. We post it as supplied by the authors.

Supplement to: Chemaitelly H, Ayoub HH, Omori R, et al. HIV incidence and impact of interventions among female sex workers and their clients in the Middle East and north Africa: a modelling study. *Lancet HIV* 2022; **9:** e496–505.

Appendix

Table of Contents

Box S1: **Search terms**......3

Section S1. Details of the mathematical modelling methods......5

Heterosexual sex work network......5

HIV sexual transmission in FSW-client partnerships......6

HIV transmission through drug injection......6

HIV sexual transmission from clients to their spouses......7

HIV natural history......8

Data sources and model parameters......8

Model simulations......9

Model fitting......10

Outcome measures......11

Oversight......12

Table S1: **Values of model parameters.**......13

Section S2. Estimation of HIV incidence in stable partners (spouses) of clients of FSWs......16

Section S3. Description of supercomputing infrastructure used to conduct the model simulations......18

Section S4. Limitations and caveats......20

Figure S1: **Map of the countries included in this study. Map includes the model-estimated HIV incidence* arising in heterosexual sex work networks for the year 2020 and its contribution to total HIV incidence in the population for 12 countries with sufficient data to conduct the simulations.**......23

Table S2: **HIV epidemiological measures among FSWs, clients, and client spouses in 2020 by increasing the sizes of the simulated cohorts.** The table includes estimated epidemiological measures for Morocco, as an illustrative example.......24

Figure S2: **Model fitting for Morocco as an example of the model fitting for a country with no significant HIV transmission through injecting drug use among female sex workers (FSWs).** A) Fitting of HIV prevalence data among FSWs. B) The relationship between HIV prevalence among FSWs and the overall sexual partnership formation rate across the 50 best model fits......25

Figure S3: **Model fitting for Iran as an example of the model fitting for a country with significant HIV transmission through injecting drug use among female sex workers (FSWs).** A) Fitting of HIV prevalence data among FSWs. B) Fitting of HIV prevalence data among FSWs who inject drugs. C) The relationship between HIV prevalence among FSWs and the overall sexual partnership formation rate across the 50 best model fits. D) The relationship between HIV prevalence among FSWs who inject drugs and the hazard rate of acquiring HIV through injecting drug use across the 50 best model fits......26

Figure S4: **Contribution of HIV incidence occurring in heterosexual sex work networks to total HIV incidence in MENA.**......27

Table S3: **Estimates of the number and proportion of HIV infections averted over 10 years by increasing the coverage of select interventions among FSWs in MENA.** This table includes results for countries with no significant injecting drug use among FSWs. Baseline coverage was used whenever it was higher than that set in the investigated scenario. 28

Table S4: **Estimates of numbers and proportions of HIV infections averted over 10 years by increasing the coverage of select interventions among FSWs in MENA.** This table includes results for countries with significant injecting drug use among FSWs. Baseline coverage was used whenever it was higher than that set in the investigated scenario.. 30

References... 31

Box S1: Search terms

PubMed

Sex work

"Extramarital Relations"[Mesh] OR “Sex Work*”[Mesh] OR "Sex/analysis"[Mesh] OR "Sex/statistics and numerical data"[Mesh] OR "Sexual partners"[Mesh] OR "Sex Trafficking/epidemiology"[Mesh] OR "Sex Trafficking/statistics and numerical data"[Mesh] OR Sex work*[Text] OR Sexual work*[Text] OR Sexwork*[Text] OR Sex-work*[Text] OR Sexual partner*[Text] OR Sex partner*[Text] OR Sexual contact*[Text] OR FSW[Text] OR FSWs[Text] OR CSW[Text] OR CSWs[Text] OR SW[Text] OR SWs[Text] OR TSW[Text] OR TSWs[Text] OR TS[Text] OR Travailleuse* sexe[Text] OR Travailleuse* sex[Text] OR Bar girl*[Text] OR Callgirl*[Text] OR Call girl*[Text] OR Escort*[Text] OR Masseuse*[Text] OR Hostess*[Text] OR ((Premarital[Text] OR Pre-marital[Text] OR Pre marital[Text] OR Extramarital[Text] OR Extra-marital[Text] OR Extra marital[Text] OR Illicit[Text] OR Illegal[Text]) AND (Sex[Text] OR Sexual[Text] OR Relation*[Text])) OR Outside marriage[Text] OR Out of marriage[Text] OR “Illegal social behavior”[Text] OR “Illegal social behaviour”[Text] OR Adultery[Text] OR Prostitut*[Text] OR Promiscu*[Text] OR Female entertain*[Text] OR Sex entertain*[Text] OR Sexual* entertain*[Text] OR Entertainment work*[Text] OR Sex industr*[Text] OR Sex establishment*[Text] OR Brothel*[Text] OR Red light[Text] OR Red-light[Text] OR Red district*[Text] OR Nightclub*[Text] OR Pimp[Text] OR ((Intergenerational[Text] OR Cross-generation*[Text] OR Cross-generational[Text] OR Recreational[Text] OR Commercial[Text] OR Transaction*[Text] OR Casual[Text] OR Group[Text] OR Informal[Text] OR Street[Text] OR Migrant*[Text] OR Survival[Text] OR Occupational[Text] OR Tourism[Text]) AND (Sex[Text] OR Sexual*[Text])) OR Sex seeking[Text] OR Sex-seeking[Text] OR Solicit*[Text] OR ((Provision*[Text] OR Provider*[Text] OR Provid*[Text] OR Sell*[Text] OR Sold[Text] OR Exchang*[Text] OR Trad*[Text] OR Favor*[Text] OR Consum*[Text] OR Commodi*[Text] OR Paid[Text] OR Paying[Text] OR Pay[Text] OR Payer*[Text] OR Buying[Text] OR Buy[Text] OR Buyer*[Text] OR Charg*[Text] OR Engag*[Text] OR Service*[Text] OR Money[Text] OR Cash[Text] OR Drug*[Text] OR Goods[Text] OR Gift*[Text]) AND (Sex[Text] OR Sexual*[Text])) OR Hidden population*[Text] OR Hard to reach population*[Text] OR Hard-to-reach population*[Text] OR Core group*[Text] OR Core risk group*[Text] OR Vulnerable women[Text] OR Vulnerable population*[Text] OR Vulnerable female*[Text] OR Most-at-risk population*[Text] OR Most at risk population*[Text] OR High risk population*[Text] OR High-risk population*[Text] OR Population* at high risk[Text] OR Population* at high-risk[Text] OR ((Traffick*[Text] OR Slave*[Text] OR Coerc*[Text] OR Abduct*[Text] OR Exploit*[Text] OR Abuse*[Text] OR Violence[Text]) AND (Sex[Text] OR Sexual*[Text]))

MENA

"Middle East"[Mesh] OR "Islam"[Mesh] OR "Arabs"[Mesh] OR "Arab World"[Mesh] OR "Africa, Northern"[Mesh] OR "Sudan"[Mesh] OR "Somalia"[Mesh] OR "Djibouti"[Mesh] OR "Pakistan"[Mesh] OR “South Sudan”[Mesh] OR "Middle East*"[Text] OR "Middle-East"[Text] OR "North Africa*"[Text] OR "North-Africa"[Text] OR "EMRO"[Text] OR "Eastern Mediterranean"[Text] OR “Arab*”[Text] OR “Arab World”[Text] OR "Islam*"[Text] OR "Afghanistan"[Text] OR "Afghan*"[Text] OR "Algeria*"[Text] OR "Bahrain*"[Text] OR "Djibouti"[Text] OR "Egypt*"[Text] OR "Jordan*"[Text] OR "Kuwait*"[Text] OR "Lebanon"[Text] OR "Leban*"[Text] OR "Libya*"[Text] OR "Iran*"[Text] OR "Iraq*"[Text] OR "Morocco"[Text] OR “Moroccan*”[Text] OR "Oman*"[Text] OR "Pakistan*"[Text] OR "Qatar*"[Text] OR "Saudi*"[Text] OR "Somalia"[Text] OR "Somal*"[Text] OR "Sudan*"[Text] OR "Syria*"[Text] OR "Tunisia*"[Text] OR "United Arab Emirates"[Text] OR "Emirat*"[Text] OR "West Bank"[Text] OR "Ghaza*"[Text] OR "Gaza*"[Text] OR "Palestine"[Text] OR "Palestinian*"[Text] OR "Yemen*"[Text] OR “UAE”[Text] OR “KSA”[Text]

Women

"Female/analysis"[Mesh] OR "Female/statistics and numerical data"[Mesh] OR “Women/epidemiology”[Mesh] OR “Women/statistics and numerical data”[Mesh] OR Women[Text] OR Girl*[Text] OR Female*[Text]

HIV

"HIV"[Mesh] OR "HIV Seroprevalence"[Mesh] OR "HIV Seropositivity"[Mesh] OR "HIV Infections"[Mesh] OR "Acquired Immunodeficiency Syndrome"[Mesh] OR "AIDS Serodiagnosis"[Mesh] OR "HIV Antibodies"[Mesh] OR HIV[Text] OR HIV/AIDS[Text]

FINAL PUBMED SEARCH
“Sex work” AND “MENA” AND “Women” AND “HIV”

Embase

Sex work

exp prostitution/ or exp casual sex/ or exp transactional sex/ or exp group sex/ or exp sex tourism/ or exp sexual promiscuity/ or exp extramarital sex/ or exp premarital sex/ or exp sexual relation/ or exp sexual partners/ or ((exp sex trafficking/ or exp sexual exploitation/ or exp sexual coercion/) NOT Child) or (sex* work* or sexwork* or sex-work* or sex partner* or sexual partner* or sexual contact* or premarital sex or premarital sexual or premarital relation* or pre-marital sex or pre-marital sexual or pre-marital relation* or pre marital sex or pre marital sexual or pre marital relation* or extramarital sex or extramarital sexual or extramarital relation* or extra-marital sex or extra-marital sexual or extra-marital relation* or extra marital sex or extra marital sexual or extra marital relation* or illicit sex or illicit sexual or illicit relation* or illegal sex or illegal sexual or illegal relation* or (out* ADJ1 marriage) or illegal social behavio?r or adultery or prostitut* or promiscu* or

FSW or FSWs or CSW or CSWs or SW or SWs or TSW or TSWs or TS or (women ADJ4 sex*) or (Travailleuse* ADJ1 sex*) or bar girl* or call girl* or callgirl* or escort* or masseuse* or hostess* or female entertain* or sex entertain* or sexual entertain* or entertainment work* or sex industr* or sex establishment* or brothel* or red light or red-light or (red ADJ1 district*) or nightclub* or pimp or recreation* sex* or intergenerational sex* or cross-generation sex* or cross-generational sex* or commercial sex* or transactional sex* or sex* transaction* or casual sex* or informal sex* or group sex* or street sex* or (migra* ADJ4 sex*) or (sex* ADJ4 migra*) or survival sex* or occupational sex* or sex* tourism or sex seeking or sex-seeking or solicit* or (consum* ADJ4 sex*) or (sex* ADJ 4 consumer) or (sex* ADJ4 consumers) or (sex* ADJ4 provi*) or (provi* ADJ4 sex*) or (sell* ADJ4 sex*) or (sex* ADJ4 sell*) or sold sex* or (exchang* ADJ4 sex*) or (sex* ADJ4 exchange) or (trading ADJ4 sex*) or (trade* ADJ4 sex*) or sex* trade or sex* favor* or (commodi* ADJ4 sex*) or (sex* ADJ4 commodi*) or (paid ADJ4 sex*) or (pay* ADJ4 sex*) or (sex* ADJ4 pay*) or (buy* ADJ4 sex*) or (sex* ADJ4 buy*) or (charg* ADJ4 sex*) or (sex* ADJ4 charg*) or (engag* ADJ4 sex*) or (sex* ADJ4 engage*) or (sex* ADJ4 service*) or (service* ADJ4 sex*) or (money ADJ4 sex*) or (sex* ADJ4 money) or (cash ADJ4 sex*) or (sex* ADJ4 cash) or (sex* ADJ4 drug*) or (drug* ADJ4 sex*) or (sex* ADJ4 goods) or (goods ADJ4 sex*) or (sex* ADJ4 gift*) or (gift* ADJ4 sex*) or hidden population* or hard to reach population* or hard-to-reach population* or (core ADJ1 group*) or vulnerable women or vulnerable female*).mp. or ((vulnerable population* or most-at-risk population* or most at risk population* or high risk population* or high-risk population* or population* at high risk or population* at high-risk).mp. AND (sex* or infection* or STI or STIs or STD or STDs or human immunodeficiency virus or HIV* or AIDS* or acquired immune deficiency syndrome or acquired immunodeficiency syndrome).mp.) or ((sex trafficking or sexual trafficking or (traffick* ADJ4 sex*) or sex* slave* or sex* coerc* or sex* abduct* or sex* exploit* or sex* abuse* or sex* violence) NOT Child).mp. or ((women ADJ4 traffick*) or (girls ADJ4 traffick*) or (female* ADJ4 traffick*) or (traffick* ADJ4 women) or (traffick* ADJ4 girls) or (traffick* ADJ4 female*)).mp.
MENA
exp Middle East/ or exp North Africa/ or exp Arab/ or exp Afghanistan/ or exp Djibouti/ or exp Pakistan/ or exp Somalia/ or exp Sudan/ or exp South Sudan/ or Middle East.mp. or North Africa.mp. or EMRO.mp. or Eastern Mediterranean.mp. or Arab.mp. or Arabs.mp. or Arab World.mp. or Islam.mp. or Afghanistan.mp. or Afghan*.mp. or Algeria*.mp. or Bahrain*.mp. or Djibouti.mp. or Egypt*.mp. or Jordan*.mp. or Kuwait*.mp. or Leban*.mp. or Libya*.mp. or Iran*.mp. or Iraq*.mp. or Morocc*.mp. or Oman*.mp. or Pakistan*.mp. or Qatar*.mp. or Saudi*.mp. or Somal*.mp. or Sudan*.mp. or Syria*.mp. or Tunisia*.mp. or United Arab Emirates.mp. or Emirat*.mp. or West Bank.mp. or Ghaza*.mp. or Gaza*.mp. or Palestin*.mp. or Yemen*.mp. or UAE.mp. or KSA.mp.
Women
exp female/ or (women or girl* or female*).mp.
HIV
exp Human immunodeficiency virus/ OR exp Human immunodeficiency virus infection/ OR exp acquired immune deficiency syndrome/ OR exp acute HIV infection/ OR exp Human immunodeficiency virus prevalence/ OR exp Human immunodeficiency virus antibody/ OR HIV.mp. OR AIDS Serodiagnosis.mp.
**FINAL EMBASE SEARCH**<br>**“Sex work” AND “MENA” AND “Women” AND “HIV”**

Abbreviations: *FSWs* female sex workers

Section S1. Details of the mathematical modelling methods

Heterosexual sex work network

In the model, each female sex worker (FSW) or client in the network enters/exits the sexual network, forms/dissolves sexual partnerships, or acquires HIV through sex or by injecting drugs at event-specific probabilities at each time step in each simulation run. The sexual network is constructed assuming that the number of sexual partnerships formed by each regular or non-regular client with FSWs follows a gamma distribution, reflecting sexual network and behavior studies.[1-6] The mean and variance (at 25% of the mean) of these distributions were informed by country-level data on sexual behavior in heterosexual sex work networks (HSWNs) for each of regular and non-regular clients, with higher mean and variance for non-regular clients.[1] Each month, every regular or non-regular client may form a new partnership with one or more FSWs, based on a random probability drawn from these distributions. Existing partnerships may also dissolve stochastically assuming an exponential distribution at a rate of inverse of duration of partnerships, which varies based on whether they involve a regular or non-regular client. Accordingly, in such sexual networks, each client randomly selects FSW partners, but clients may have different propensities to form partnerships, a situation known as proportionate mixing.[5,7]

FSWs exit the HSWN if they cease to practice sex work, and for clients if they cease seeking sex with FSWs, or through natural and AIDS-related mortality (table S1). Lower HIV transmission, slower AIDS disease progression, and higher life expectancy were assumed for individuals on antiretroviral therapy (ART; table S1). Those who exit the HSWN are replaced by susceptible persons, thus maintaining a fixed cohort size for FSWs and clients.

While the model assumes that HIV acquisition among FSWs can occur through sex with a client or through injecting drug use with an injecting partner, HIV acquisition among clients was assumed to occur only through sex with an HIV-positive FSW. Other sources of infection, such as the client's spouse, other heterosexual partners, male same-sex partners, and injecting drug use were not considered. Evidence suggests that the risk of HIV infection through these modes of exposure among clients is probably substantially smaller than the risk of infection through sex with a FSW in most countries of the Middle East and North Africa (MENA).[1,8-10]

HIV sexual transmission in FSW-client partnerships

Probability of HIV sexual transmission in an HIV sero-discordant partnership, that includes an HIV-positive FSW/client and a susceptible counterpart, was determined from the probability of transmission per coital act per HIV stage of infection, number of coital acts per partnership, which varied based on whether partnerships were with regular or non-regular clients, and interventions that affect HIV transmission.

These interventions included ART among FSWs and their injecting partners, condom use in the partnership, male circumcision in the client, and pre-exposure prophylaxis (PrEP) in the FSW. Coverage of these interventions for FSWs and clients was based on data for each country and was implemented in the model by random assignment.

HIV transmission through drug injection

Proportions of FSWs who inject drugs were based on data for each country. Since the injecting partners of FSWs can be other FSWs or persons external to the modeled sexual network of FSWs and clients, HIV acquisition through injecting drug use was modeled through an external hazard rate (force of infection) that depended on whether the FSW was on PrEP and whether her

injecting partner was on ART. Otherwise, a constant hazard rate was assumed and was derived by fitting model output to country-level data on HIV prevalence among FSWs who inject drugs,[1] or alternatively if such data were not available, to HIV prevalence among people who inject drugs (PWID).[11] FSWs who inject were assumed to inject for a specific duration, set at 10 years,[11] which differed from the duration of sex work set at 35 years.[1] Effect of PrEP on injecting drug use transmission was assumed to be equivalent to that for sexual transmission.

The proportion of FSWs who inject drugs (table 2 of main text) is ≥10-fold higher than that among clients and spouses, assuming that the latter proportion can be approximated by that among the wider population.[11,12] Therefore, HIV acquisition through injecting drug use was not modeled among clients and spouses at it likely plays a much less significant role in transmission dynamics among them.

HIV sexual transmission from clients to their spouses

HIV sexual transmission from clients to their spouses was modeled using a separate deterministic model, but using the individual-based model output as input (section S2). This was done for computational efficiency and because the stochastic and non-linear effects are less likely to be critical here, as the transmission is one-directional from clients to their spouses. Numbers of HIV transmissions from clients to spouses were estimated using the proportion of clients in spousal partnerships, HIV prevalence among clients, numbers of susceptible spouses, and probability of HIV transmission per partnership. The latter was estimated using the probability of transmission per coital act per HIV stage of infection, numbers of coital acts per partnership, condom use, and ART coverage among clients. It was assumed that all HIV incidence among spouses occurs through transmission from the HIV-positive client to the susceptible spouse, as other sources of exposure are likely limited in the MENA context.[1,8-10]

HIV natural history

HIV natural history was based on established empirical epidemiological measures (table S1). Progression through each of HIV infection stages was modeled assuming an exponential distribution through rates derived as the inverse of duration of each HIV stage and implemented through a stochastic process.

Data sources and model parameters

The primary data source for this modeling study was the recently completed systematic review of HIV, sexual and injecting behavior, and population size estimates in FSWs and clients in MENA.[1] Countries were included in the present study if they had sufficient input data to simulate the HIV epidemic in the HSWN *and* HIV prevalence among FSWs was ≥0.5%. Otherwise, it was not feasible to conduct the simulations. Twelve of the 23 MENA countries were included: Algeria, Bahrain, Djibouti, Iran, Libya, Morocco, Pakistan, Somalia, South Sudan, Sudan, Tunisia, and Yemen (figure S1). Of note, that we included South Sudan because it was historically part of the World Health Organization (WHO) and the Joint United Nations Programme on HIV/AIDS (UNAIDS) definitions for this region and because available studies for Sudan before 2011, the year of independence of South Sudan, included data from both Sudan and the newly indpendent Republic of South Sudan. Injecting drug use among FSWs was modelled in countries in which evidence suggested a significant role for injecting drug use in the HIV epidemic.[1] These included Bahrain, Iran, Libya, and Pakistan.

Country-specific parameter values were selected based on the most recent representative studies identified through the aforementioned systematic review.[1] Priority was given to studies with rigorous sampling methodologies, such as integrated bio-behavioral surveillance (IBBS) surveys. Where several nationally representative estimates based on IBBS surveys were available,[1] the

mean of these estimates was considered. Otherwise, data collected after the year 2000 were pooled using random-effects meta-analysis. This methodology used Freeman-Tukey type arcsine square-root transformation to stabilize variances[13,14] before weighting measures using the inverse-variance method,[14,15] followed by pooling using DerSimonian-Laird random-effects models to account for sampling variation and true heterogeneity.[16,17] Data for coverage of interventions were primarily based on findings of the systematic review,[1] or alternatively, on UNAIDS compilations,[18] or imputed using the regional median for these parameters.[1]

Demographic and Health Survey data on men in the general population were used to derive, for each country, the proportion of clients in spousal partnerships (defined as a marital/cohabiting partnership for ≥1 year) and the proportion of sexual acts protected by condom use in these partnerships.[19] The proportion of FSWs in spousal partnerships was based on the extracted data of the systematic review of sexual behavior of FSWs.[1] For countries with missing information, measures were imputed by pooling regional data using random-effects meta-analysis.

The population size of FSWs and clients in each country was based on country-level data.[1] Other model parameters, such as for HIV transmission and efficacy/effectiveness and coverage of interventions, were based on current evidence in the literature (table S1 and tables 1-2 of main text).

Model simulations

The model-generated sexual network was established with a “burn-in” of 50 years to ensure equilibrium of network structure prior to HIV introduction. Subsequently, HIV infection was seeded and the model was run for an additional “burn-in” of 300 years to ensure epidemic equilibrium in each country by 2020. These long durations of burn-in were chosen to ensure the stability of the epidemic simulations. Shorter durations could have been chosen, but we opted for

caution to ensure no temporal effects bias our estimates. Since epidemiological measures of interest, such as HIV incidence, were estimated over a short time horizon of one year, and in absence of quality country-level trend data for HIV prevalence in FSWs and clients in nearly all MENA countries,[1] analyses were implemented starting from this epidemic equilibrium.

Model predictions for each country were based on the mean and 95% uncertainty intervals (UIs) of distributions of outcome measures generated by 500 simulation runs. At this number of runs, the mean and distribution of outcome measures minimally varied with an increase in the number of simulations. The 95% UIs were generated directly from these distributions of simulation runs after excluding runs with HIV stochastic extinction. Extinctions were excluded because they resulted from the finite size of the simulated sexual network (stochastic fade-out), but unlikely to occur in reality considering that the actual size of the sexual network is much larger. For computational efficiency, after experimenting with different cohort sizes, simulations were performed using a cohort of 600 FSWs and 6,000 clients (one-third of which are regular and two-thirds are non-regular/one-time clients), as informed by MENA data.[1] Outcome measures were subsequently scaled-up to reflect the actual population sizes in each country.[1]

Model fitting

Model fitting to HIV prevalence data among FSWs and HIV prevalence among FSWs who inject drugs was performed to estimate the overall sexual partnership formation rate and the baseline hazard rate of acquiring HIV through injecting drug use in each included country. Nonlinear least-square fitting using the Nelder-Mead simplex algorithm[20] was implemented iteratively to generate a set of 50 best model fits. A best model fit was defined as a relative error of <5% between model predictions and empirical data. The final best model fit was the most probable value for the sexual partnership rate and injecting hazard rate among the 50 best model fits—

taking instead the means or the medians resulted in inferior fits. Examples of the model fitting for Morocco (a country with no significant HIV transmission through injecting drug use among FSWs) and Iran (a country with significant HIV transmission through injecting drug use among FSWs), are shown in figures S2 and S3, respectively.

This process for model fitting, and the model's ability to generate model output consistent with empirical data and our understanding of the epidemiology, provide a validation of this model. For further validation, an array of sensitivity analyses was conducted as part of the model development to ensure consistency of the model and its outcomes. Means of model outcomes were also compared to several deterministic models that were designed to capture the structure of this individual-based model.

Outcome measures

HIV epidemiological measures

HIV incidence was defined as the number of new infections per year and was calculated by summing new infections occurring among FSWs (or clients) at each time-step (1 month) during the year. HIV incidence rate was defined as the number of new infections per 1,000 (susceptible) person-years and was calculated by dividing the number of incident infections among FSWs, clients, and client spouses by the respective numbers of susceptible individuals in these populations at the start of that year. The relative contribution of sexual versus injecting HIV acquisitions to total incidence among FSWs was estimated by dividing the number of incident infections resulting from each of sexual and injecting transmission during one year by all incident infections during that year. The relative contribution of HSWNs to HIV incidence in the total adult population was estimated by dividing the sum of incident infections arising among

FSWs, clients, and client spouses over the duration of a year, by the total HIV incidence in the population (15-49 years) during that year, as estimated by UNAIDS.[18]

Impact of interventions

The impact of expanding HIV interventions among FSWs on HIV incidence arising in HSWNs was assessed by estimating, using 500 simulation runs, the mean number of infections that would be averted over a 10-year duration after implementing the interventions, and the proportional decrease in incidence during this time (table 3 of main text).

Oversight

Ethical approval was not needed for this modeling study because the study uses published data for model input and provides aggregate measures for HIV incidence and impact of interventions.

Table S1: **Values of model parameters.**

Parameter	Value	Justification/Source
**HIV transmission and natural history**		
Transmission probability per coital act		
Acute stage of HIV infection	0.0360	Observational cohorts and subsequent analyses.[21,22]
Latent stage of HIV infection	0.0008	Observational cohorts and subsequent analyses.[21,22]
Advanced stage of HIV infection	0.0042	Observational cohorts and subsequent analyses.[21,23-26]
From clients to stable sexual partners (spouses)	0.0018	Weighted average derived using transmission probability per coital act for each HIV infection stage and time spent in that stage.
Duration of HIV infection stages in absence of ART		
Acute stage of HIV infection	49 days	Observational cohorts and subsequent analyses.[21,22,27-32]
Latent stage of HIV infection	9 years	Observational cohorts and subsequent analyses.[21,22,27-32]
Advanced stage of HIV infection	2 years	Observational cohorts and subsequent analyses.[21,22,26-32]
**HIV prevalence**		
FSWs	See Tables 1-2	Based on findings of FSWs in MENA systematic review.[1]
FSWs who inject drugs	See Table 2	Based on findings of FSWs in MENA systematic review, in countries where evidence suggests a significant role for injecting drug use in the HIV epidemic.[1] For countries with missing information, findings were based on PWID in MENA systematic review,[11] or UNAIDS data.[18]
Clients of FSWs	See Tables 1-2	Model prediction.
Client spouses	See Tables 1-2	Assumed to be 1/3 of HIV prevalence in clients of FSWs.[9,10,33]
**Population size**		
FSWs	See Tables 1-2	Based on findings of FSWs in MENA systematic review.[1] For countries with missing information, findings were based on median proportion of reproductive-age women reporting current/recent sex work across MENA countries (0.6%, median out of 111 studies) in FSWs in MENA systematic review,[1] and estimates for the size of the population of adult women aged 15-49.[34]
Clients of FSWs	See Tables 1-2	Assumed to be ten times larger than the size of the FSWs population based on FSWs in MENA systematic review[1] and modeling studies.[9,10]
**Sexual risk behavior**		
Number of coital acts with a FSW		
Regular clients	3 acts per month	Based on findings of FSWs in MENA systematic review.[1]
One-time clients	1 act per month	Based on findings of FSWs in MENA systematic review.[1]
Partnership duration with a FSW		
Regular clients	3 months	Reasonable value informed by findings of FSWs in MENA systematic review.[1]
One-time clients	1 month	Reasonable value informed by findings of FSWs in MENA systematic review.[1]
Proportion of clients in stable partnerships		
Morocco	52.3%*	Demographic and Health Survey (2003).[19]
Yemen	61.2%*	Demographic and Health Survey (2003).[19]
Pooled estimate-MENA countries with data†	56.4%	Demographic and Health Surveys.[19]
Number of coital acts with spouses for regular and one-time clients	25 acts per year	Reasonable value considering that over 80% of women seeking antenatal or family planning services had sexual relations at least once per week[35] and accounting for the fact that clients of FSWs have reduced number of acts with spouses.
Overall rate of sexual partnership formation	Model fitted	This country-specific model parameter was derived by fitting model output to country-level data on HIV prevalence among FSWs.

**Injecting risk behavior**		
Proportion of FSWs who inject drugs	See Table 2	Median of country-specific estimates based on findings of FSWs in MENA systematic review.[1] For countries with missing information, findings were based on most representative estimates based on findings of a systematic review of HIV among PWID in MENA and recent unpublished updates.[11]
Time spent in injecting drug use	10 years	Based on findings of systematic reviews.[11,36]
Hazard rate of acquiring HIV through injecting drug use	Model fitted	This country-specific hazard rate was derived by fitting model output to country-level data on HIV prevalence among FSWs who inject drugs, or alternatively if such data were not available, to HIV prevalence among PWID.
**HIV prevention interventions**		
ART		
Efficacy in preventing HIV transmission to partners	96%	Based on findings of a randomized clinical trial.[37]
Real-world effectiveness in achieving viral suppression in FSWs	57%	Based on findings of a global systematic review,[38] as no MENA-specific estimate was available. This might be a conservative estimate given recent improvements in ensuing viral load suppression among those receiving ART.
Effectiveness in slowing disease progression from the latent to the advanced stage of HIV infection	1/3	Based on findings of cohort and modeling studies.[39-41]
Effectiveness in slowing disease progression to AIDS death for those in the advanced stage of HIV infection	1/3	Based on findings of cohort and modeling studies.[39-41]
Coverage in clients/PLHIV	See Tables 1-2	UNAIDS[18] and World Bank[42] data.
Coverage in FSWs	See Tables 1-2	UNAIDS[18] and World Bank[42] data. Coverage was assumed to be equal to that estimated for all PLHIV as no recent data on coverage among FSWs was available (except for South Sudan[1]).
Condoms		
Effectiveness in reducing HIV transmission	80%	Based on findings of observational studies.[43-45]
Coverage in commercial sex	See Tables 1-2	Median of country-specific estimates based on findings of FSWs in MENA systematic review.[1] For countries with missing information, findings were based on median proportion of FSWs reporting condom use at last sex (44.0%, median out of 97 studies) in FSWs in MENA systematic review.[1]
Coverage in spousal partnerships[†]		
Morocco	1.5%	Demographic and Health Survey (2003).[19]
Pakistan	10.6%	Demographic and Health Survey (2017).[19]
Yemen	0.5%	Demographic and Health Survey (2003).[19]
Pooled estimate-MENA countries with data[‡]	2.9%	Demographic and Health Surveys.[19]
Infant male circumcision or VMMC		
Efficacy in reducing HIV transmission	58%	Based on findings of clinical trials and systematic review.[46-49]
Infant male circumcision coverage	See Tables 1-2	Global male circumcision prevalence data.[50]
PrEP		
Effectiveness in reducing HIV transmission	51%	Based on findings of a systematic review.[51]
Coverage in clients	See Tables 1-2	UNAIDS data.[18]
Coverage in FSWs	See Tables 1-2	UNAIDS data.[18]

Abbreviations: ART: anti-retroviral therapy, FSW: female sex workers, MENA: Middle East and North Africa, NA: not applicable, PLHIV: people living with HIV, PrEP: pre-exposure prophylaxis, PWID: people who inject drugs, UNAIDS: The Joint United Nations Programme on HIV/AIDS, VMMC: voluntary male circumcision; WHO-EMRO: World Health Organization's Regional Office for the Eastern Mediterranean.

[*]Data only available for women, the fraction of men in spousal partnerships was assumed to be equal to that of women.

†Proportion of women reporting condoms as current contraceptive method.
‡Includes all MENA countries with data regardless of whether these countries qualified for inclusion in this study.

Section S2. Estimation of HIV incidence in stable partners (spouses) of clients of FSWs

We modelled HIV sexual transmission from clients of FSWs to their stable partners (spouses) using a deterministic modelling component the input of which was provided by the output of the individual-based model of FSWs and clients. This deterministic component applies only to the spouses of clients. The transmission dynamics among FSWs and clients are fully described using the individual-based model described in section S1.

All incidence in spouses was assumed to arise from HIV transmission from the HIV-positive client/husband to the spouse. This is supported by empirical evidence, specifically in the context of MENA, indicating limited risk of HIV acquisition for women in marital partnerships from a source outside this partnership.[8-10,52-55]

The probability of HIV transmission from an HIV-positive client (not on ART) to a susceptible spouse over the course of one year is given by

$$t_{Spouse} = 1 - \left(1 - \beta_{Spouse}\right)^{n_{Spouse}\left(1 - f_{condom}\right)\tau_{Spouse}} \left(1 - \left(1 - e_{condom}\right)\beta_{Spouse}\right)^{n_{Spouse} f_{condom}\tau_{Spouse}}$$

Here, β_{Spouse} is the weighted average for the probability of HIV transmission per unprotected coital act across the different HIV infection stages (the weighted average is given by the sum of the product of HIV transmission probability per unprotected coital act in a specific HIV infection stage by the duration spent in that stage relative to the total duration of infection), n_{Spouse} is the number of coital acts in the spousal partnership over the course of a year, f_{condom} is the fraction of acts protected by condom use, e_{condom} is the effectiveness of condom use in reducing HIV transmission, and τ_{Spouse} is the duration of follow-up (here, assumed to be one year).

The number of HIV sero-discordant spousal partnerships for each of regular and non-regular clients is given by

$$N_{disc} = NF_{Marital}P_{Client}\left(1-P_{Spouse}\right)$$

Here, N is the total number of regular or non-regular clients of FSWs, $F_{Marital}$ is the fraction of clients in spousal partnerships (assumed to be the same for regular and non-regular clients), P_{Client} is HIV prevalence among regular or non-regular clients of FSWs, and P_{Spouse} is HIV prevalence among spouses (assumed to be one third of that among clients of FSWs[9,10,33]).

For spouses of each of regular or non-regular clients of FSWs, HIV incidence is hence given by

$$I_{Spouse} = N_{disc}t_{Spouse}\left(1-e_{ART}Coverage_{ART}\right)$$

Here, e_{ART} is the effectiveness of ART in reducing HIV transmission from an HIV-positive client to the spouse and $Coverage_{ART}$ is the coverage of ART among clients.

HIV incidence rate is thus given by:

$$IR = \frac{I_{Spouse}}{(1-P_{Spouse})NF_{Marital}}.$$

Section S3. Description of supercomputing infrastructure used to conduct the model simulations

Simulations were conducted using the Red Cloud infrastructure of Cornell University (https://www.cac.cornell.edu/services/cloudservices.aspx). Red Cloud is a subscription-based infrastructure as a service cloud that provides root access to virtual servers and storage on-demand at Cornell University in New York, USA. One Red Cloud subscription provides 8,585 core hours (approximately one year running a single-core instance) and includes 50GB storage. Additional storage can be purchased separately. Each subscription can be used as fast or as slowly as needed, and never expires.

Users can request instances with up to 28 cores and 224 GB of RAM. Each instance is a virtual machine (VM) that deploys in a few seconds in the cloud. A variety of VM configurations as well as NVIDIA Tesla V100 and T4 GPU are available. An Intel Xeon processor core is behind each core on the virtual server for fast and consistent performance.

The Red Cloud service provided by the Cornell Center for Advanced Computing comprises an on-premise OpenStack cloud computing platform with 1400 compute cores for Infrastructure-as-a-Service (IaaS) computing. The service provides users with the ability to create and manage virtual machines from 1-28 cores, with 8GB memory per core (up to 224GB memory). The system provisions cores on a one-to-one basis, in order to provide full utilization of processor core capability. Red Cloud also provides access to 4 NVIDIA V100 GPUs and 20 NVIDIA T4 GPUs. Storage for cloud instances is provided by a Ceph storage system that gives access to 2PB of storage. Storage volumes are private to each individual user or project.

CAC supports Windows and Linux instances on Red Cloud, with pre-built instances that include MATLAB Parallel Server which allows multiple instances to do MATLAB computations in

parallel, as well as instances pre-configured with GPU libraries and common applications for computational analyses.

Our sensitivity analyses indicated that a three-fold increase in the size of the simulated populations is optimal for reducing stochastic fade-out (table S2). However, generating only baseline estimates using 100 simulation runs for one country entailed running the model for 5-6 days thus undermining the feasibility of extending this increase in population sizes to all analyses.

Section S4. Limitations and caveats

Estimates were generated through mathematical modeling, and therefore should be seen as an approximation of actual reality based on our current knowledge and the available input data that parametrized the model. Analyses were possible for only 12 of 23 MENA countries with sufficient HIV prevalence, behavioral, and risk group size estimate data to apply the model. However, these 12 countries constituted 65% of the total population of MENA and included all countries where current evidence suggests significant epidemics in HSWNs.[1] Some of the input data, such as for HIV prevalence, originated from IBBS surveys or other surveys conducted in specific settings or cities using different methodologies and are of different qualities, could be outdated, and may not represent the total FSW population in a given country at the present time, thereby possibly affecting the estimates.

To minimize bias in input data, we systematically pooled these data for more robust mean estimates that account for the totality of available evidence. Some model input data were global rather than MENA-specific such as the real-world effectiveness in achieving viral suppression among FSWs.[38] Some possible effects were not included, such as sero-sorting or differences in uptake of interventions for different groups of FSWs and clients, as supporting evidence was not available to include them in the model. Effect of other sexually transmitted infections on HIV transmission probability was not also included due to conflicting evidence,[56] but such inclusion is not likely to affect the model outcomes as the effect would have been absorbed in the overall sexual contact rate.

The model did not simulate further onward HIV transmission beyond FSWs, clients, and client spouses; thus, this study may underestimate the contribution of HSWNs to total HIV incidence in the population. The model outcomes for spouses of clients were generated through the additional

deterministic modelling component, and thus do not factor stochasticity effects leading to narrower 95% UIs.

Having quality and representative longitudinal/trend data for HIV prevalence would have been optimal as it allows fitting trends in HIV prevalence. However, in absence of such country-level trend data,[1] estimates were generated assuming endemic equilibrium. This may not have had an appreciable effect on estimated epidemiological measures such as incidence, as they were generated over only one year, but may have underestimated the impact of interventions if HIV prevalence is increasing, as suggested for the MENA region.[1]

HSWNs are large and it is not feasible computationally to simulate the entire HSWN in each country using such a fine-grained, individual-based modelling approach. Moreover, a low HIV prevalence makes the epidemic less stable as small random changes in the structure of the sexual network can result in considerable change in HIV incidence. For computational feasibility and efficiency, simulations were performed using sub-cohorts of FSWs and clients that are representative of the full cohorts of FSWs and clients. Results were subsequently scaled-up to reflect actual population sizes of FSWs and clients. This reduction in simulated cohort sizes made it difficult to simulate HSWNs and sustain HIV epidemics in countries where HIV prevalence among FSWs is ≤0.5%. These countries were thus excluded from analysis (n=6). This may also have slightly underestimated HIV incidence in included countries due to finite-network effects and higher likelihood of stochastic extinction (stochastic fade-out). Sensitivity analyses were conducted where the sample sizes of the simulated cohorts were increased by 2-fold, 3-fold, and 4-fold, to investigate the effect of stochastic fade-out on simulated model outcomes. The stochastic fade-out had a minimal effect on the point estimates of the model outcomes, but the 95% UIs were narrower with the larger simulated cohorts (table S2).

The stochastic fade-out further resulted in higher stochasticity in simulations assessing the impact of interventions up to 2030. The impact was thus assessed after 30 years “burn-in” to reduce stochasticity, and then scaled back to a 10-year duration, which may have overestimated the indirect impact of interventions on onward transmission of infection. The indirect impact of interventions on incidence is slower to materialize than the direct impact. The latter, such as for condom use, is immediate the moment a condom is used in a simulated sexual partnership.

Both point estimates and uncertainty ranges were directly obtained from model simulations, and therefore may not have factored other sources of uncertainty that may exist in real world sexual networks. Uncertainty intervals around estimates of the relative contribution of HIV incidence arising in HSWNs to total HIV incidence were often too broad. This is due to the finite-network effects and stochastic fade-out, more so when the number of infections was small leading to a higher effect of stochasticity on predicted outcomes.

Figure S1: **Map of the countries included in this study. Map includes the model-estimated HIV incidence* arising in heterosexual sex work networks for the year 2020 and its contribution to total HIV incidence in the population for 12 countries with sufficient data to conduct the simulations.**

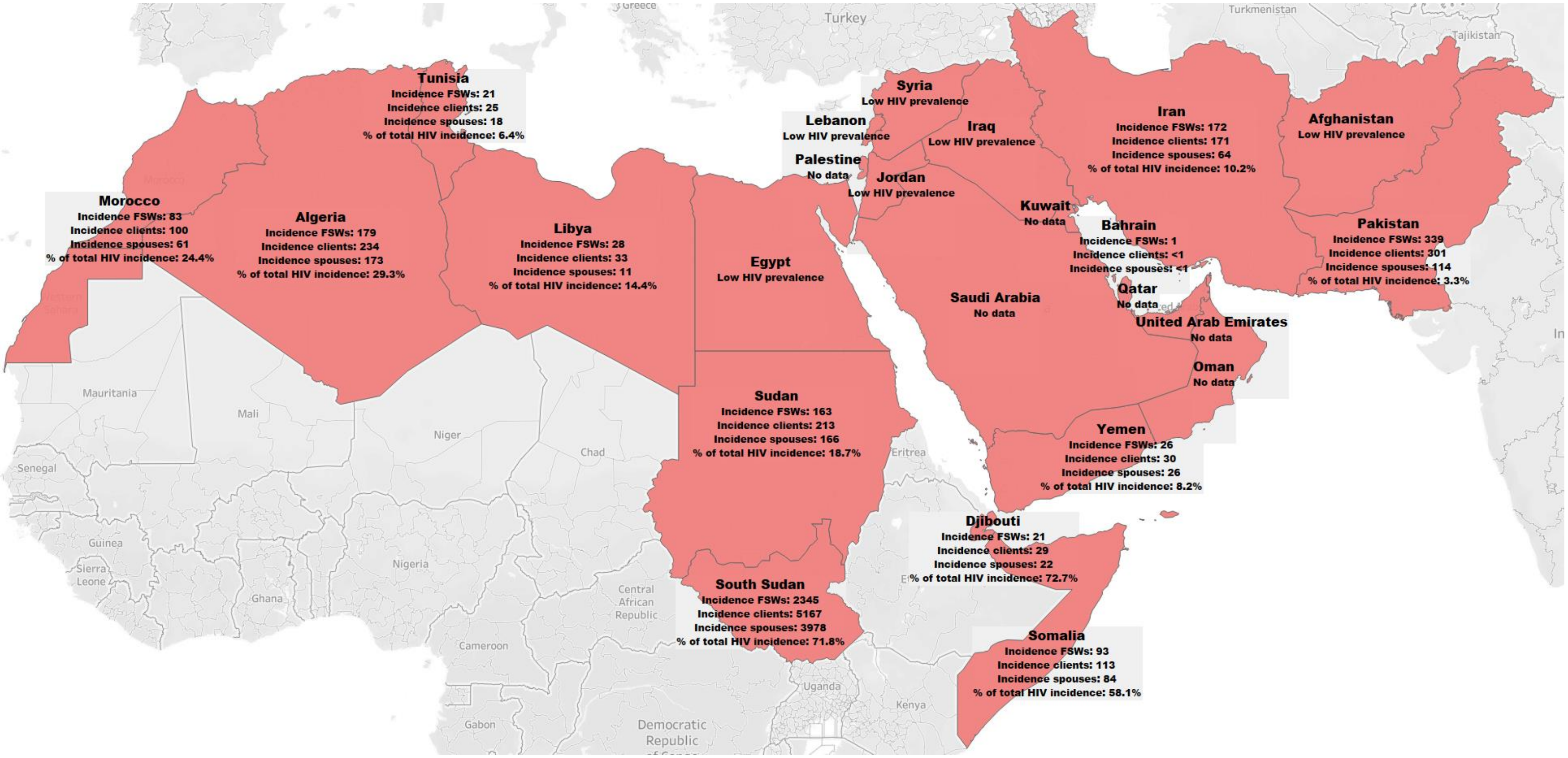

*Incidence here refers to the number of new HIV infections in 2020.

***Table S2*: HIV epidemiological measures among FSWs, clients, and client spouses in 2020 by increasing the sizes of the simulated cohorts.** The table includes estimated epidemiological measures for Morocco, as an illustrative example.

**Epidemiological measures**	**Baseline sample size**	**Two-fold the baseline sample size**	**Three-fold the baseline sample size**	**Four-fold the baseline sample size**
**Model estimates for 2020**				
**HIV prevalence**				
All FSWs (%)	2.2%	2.3%	2.4%	2.4%
95% uncertainty interval (%)	0.5-8.0%	0.4-6.5%	0.4-6.0%	0.6-5.0%
Clients of FSWs (%)	0.5%	0.6%	0.6%	0.6%
95% uncertainty interval (%)	0.1-1.9%	0.1-1.7%	0.1-1.4%	0.2-1.3%
Client spouses	0.2%	0.2%	0.2%	0.2%
95% uncertainty interval (%)	0.03-0.6%	0.03-0.6%	0.03-0.5%	0.07-0.4%
**HIV incidence in HSWNs per year (n*)**				
All FSWs (n)	83	88	91	91
95% uncertainty interval (n)	0-600	0-360	0-320	0-270
Clients of FSWs (n)	100	116	120	117
95% uncertainty interval (n)	0-600	0-420	0-360	0-300
Client spouses (n)	61	65	68	68
95% uncertainty interval (n)	11-217	13-185	14-162	19-142
**HIV incidence rate**‡ (per 1,000 person-years)				
All FSWs	1.2	1.3	1.3	1.3
95% uncertainty interval	0.0-8.7	0.0-5.2	0.0-4.7	0.0-3.9
Clients of FSWs	0.07	0.08	0.08	0.08
95% uncertainty interval	0.0-0.4	0.0-0.3	0.0-0.3	0.0-0.2
Client spouses	0.2	0.2	0.2	0.2
95% uncertainty interval	0.03-0.6	0.03-0.5	0.04-0.4	0.05-0.4

Abbreviations: FSWs: female sex workers; HSWNs: heterosexual sex work networks.
*n here refers to the number of new HIV infections expected in Morocco for the year 2020.

Figure S2: **Model fitting for Morocco as an example of the model fitting for a country with no significant HIV transmission through injecting drug use among female sex workers (FSWs).** A) Fitting of HIV prevalence data among FSWs. B) The relationship between HIV prevalence among FSWs and the overall sexual partnership formation rate across the 50 best model fits.

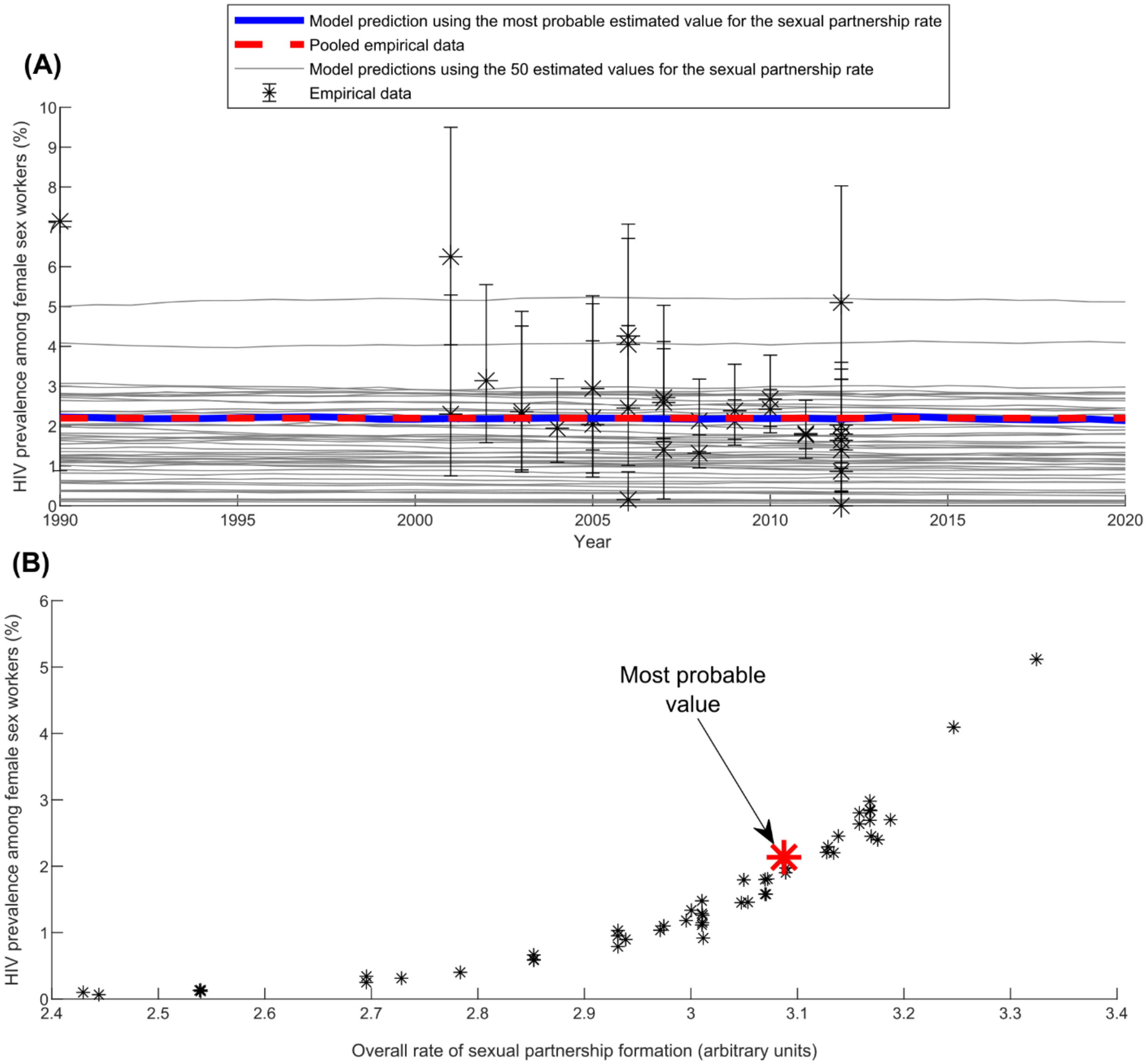

***Figure S3:* Model fitting for Iran as an example of the model fitting for a country with significant HIV transmission through injecting drug use among female sex workers (FSWs).** A) Fitting of HIV prevalence data among FSWs. B) Fitting of HIV prevalence data among FSWs who inject drugs. C) The relationship between HIV prevalence among FSWs and the overall sexual partnership formation rate across the 50 best model fits. D) The relationship between HIV prevalence among FSWs who inject drugs and the hazard rate of acquiring HIV through injecting drug use across the 50 best model fits.

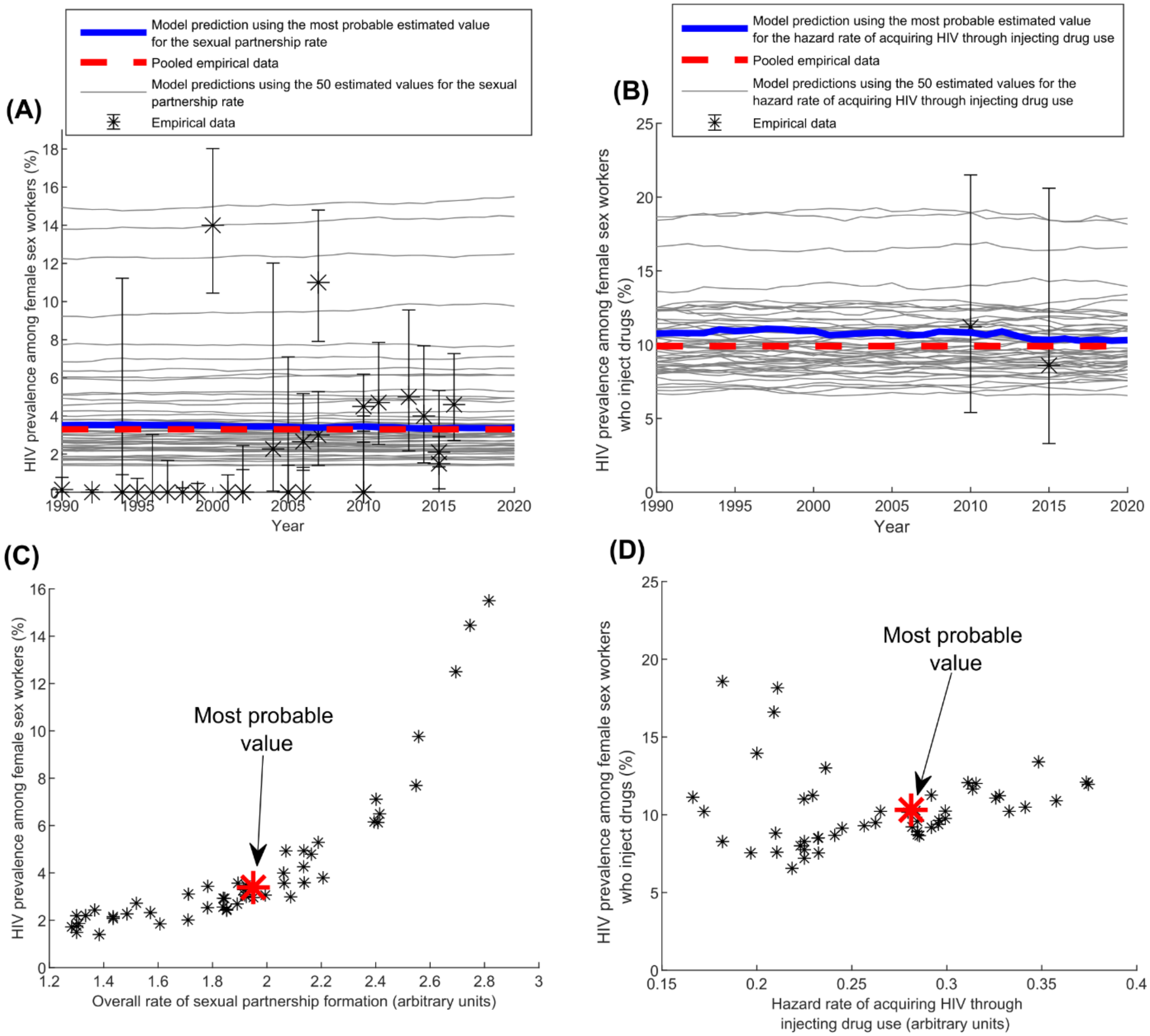

Figure S4: **Contribution of HIV incidence occurring in heterosexual sex work networks to total HIV incidence in MENA.**

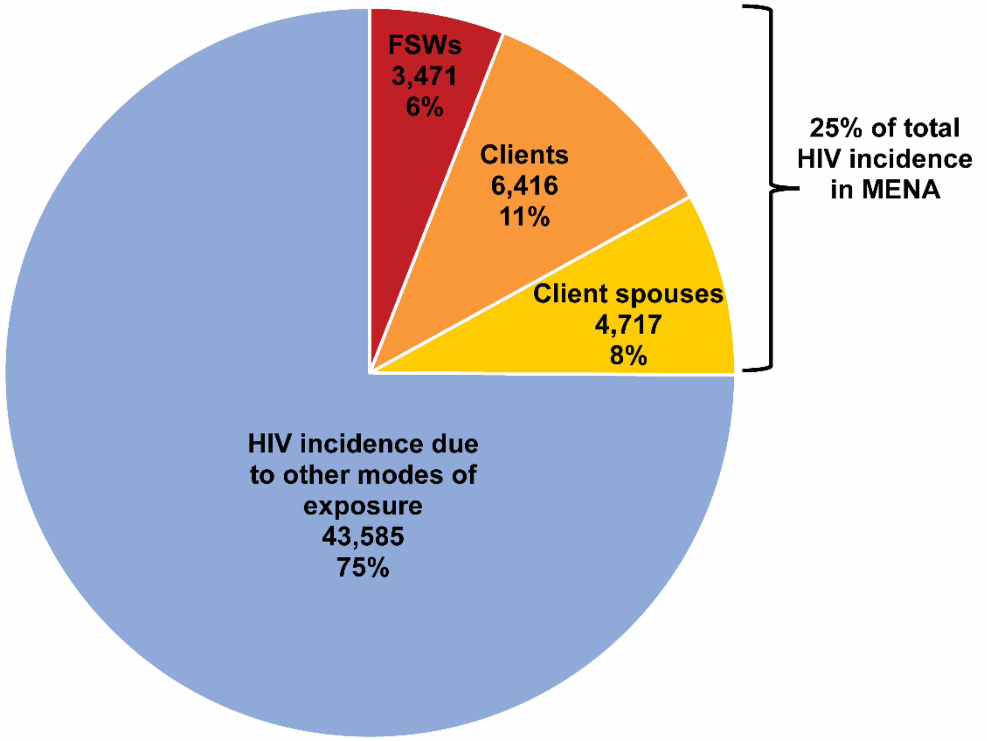

***Table S3:* Estimates of the number and proportion of HIV infections averted over 10 years by increasing the coverage of select interventions among FSWs in MENA.** This table includes results for countries with no significant injecting drug use among FSWs. Baseline coverage was used whenever it was higher than that set in the investigated scenario.

**Countries**	**Algeria**			**Djibouti**		
	**In FSWs**	**In clients**	**In client spouses**	**In FSWs**	**In clients**	**In client spouses**
**Cumulative incidence 2020-2030**	**1,905**	**2,504**	**1,809**	**234**	**308**	**229**
**Infections averted* by prevention intervention scenario-** N (%)						
ART in FSWs (eART: 0.57)						
Increasing coverage to 25%	NA	NA	NA	NA	NA	NA
Increasing coverage to 50%	118 (6.2)	338 (13.5)	110 (6.1)	20 (8.4)	47 (15.4)	17 (7.4)
Increasing coverage to 81%	429 (22.5)	1,017 (40.6)	403 (22.3)	49 (21.1)	121 (39.4)	46 (20.1)
ART in FSWs (eART: 0.96)						
Increasing coverage to 25%	NA	NA	NA	NA	NA	NA
Increasing coverage to 50%	731 (38.4)	1,193 (47.6)	489 (27.0)	82 (35.1)	144 (46.8)	56 (24.5)
Increasing coverage to 81%	1,050 (55.1)	2,010 (80.3)	826 (45.7)	121 (51.9)	244 (79.2)	98 (42.8)
Condom use (eCondom: 0.80)						
Increasing use to 50%	NA	NA	NA	NA	NA	NA
Increasing use to 80%	732 (38.4)	963 (38.5)	348 (19.2)	113 (48.3)	148 (47.9)	59 (25.8)
VMMC (eVMMC: 0.58)						
Increasing coverage to 50%	NA	NA	NA	NA	NA	NA
Increasing coverage to 80%	NA	NA	NA	NA	NA	NA
PrEP in FSWs (ePrEP: 0.51)						
Increasing coverage to 25%	348 (18.3)	251 (10.0)	107 (5.9)	25 (10.5)	6 (2.0)	-3 (-1.3)
Increasing coverage to 50%	556 (29.2)	330 (13.2)	89 (4.9)	68 (29.0)	42 (13.7)	12 (5.2)
Intervention packages						
Moderately optimistic scenario†	948 (49.8)	1,338 (53.4)	569 (31.5)	99 (42.3)	146 (47.5)	52 (22.7)
Most optimistic scenario‡	1,457 (76.5)	2,188 (87.4)	912 (50.4)	180 (77.2)	273 (88.4)	110 (48.0)
**Countries**	**Morocco**			**Somalia**		
	**In FSWs**	**In clients**	**In client spouses**	**In FSWs**	**In clients**	**In client spouses**
**Cumulative incidence 2020-2030**	**853**	**1,062**	**617**	**953**	**1,161**	**866**
**Infections averted* by prevention intervention scenario-** N (%)						
ART in FSWs (eART: 0.57)						
Increasing coverage to 25%	NA	NA	NA	NA	NA	NA
Increasing coverage to 50%	NA	NA	NA	93 (9.8)	207 (17.8)	76 (8.8)
Increasing coverage to 81%	136 (15.9)	265 (24.9)	83 (13.5)	200 (21.0)	474 (40.8)	180 (20.8)
ART in FSWs (eART: 0.96)						
Increasing coverage to 25%	NA	NA	NA	NA	NA	NA
Increasing coverage to 50%	NA	NA	NA	345 (36.2)	557 (48.0)	218 (25.2)
Increasing coverage to 81%	538 (63.1)	824 (77.6)	267 (43.3)	519 (54.5)	935 (80.6)	405 (46.8)
Condom use (eCondom: 0.80)						
Increasing use to 50%	NA	NA	NA	278 (29.1)	334 (28.8)	105 (12.1)
Increasing use to 80%	489 (57.3)	590 (55.6)	193 (31.3)	649 (68.2)	777 (66.9)	287 (33.1)
VMMC (eVMMC: 0.58)						
Increasing coverage to 50%	NA	NA	NA	NA	NA	NA
Increasing coverage to 80%	NA	NA	NA	NA	NA	NA
PrEP in FSWs (ePrEP: 0.51)						
Increasing coverage to 25%	138 (16.2)	57 (5.4)	2 (0.3)	121 (12.6)	44 (3.8)	-1 (-0.1)
Increasing coverage to 50%	325 (38.1)	235 (22.1)	80 (13.0)	235 (24.7)	102 (8.8)	9 (1.0)
Intervention packages						
Moderately optimistic scenario†	520 (61.0)	599 (56.4)	217 (35.2)	577 (60.5)	759 (65.3)	303 (35.0)
Most optimistic scenario‡	719 (84.3)	934 (88.0)	293 (47.5)	808 (84.8)	1,075 (92.5)	434 (50.1)
**Countries**	**South Sudan**			**Sudan**		
	**In FSWs**	**In clients**	**In client spouses**	**In FSWs**	**In clients**	**In client spouses**

**Cumulative incidence 2020-2030**	**24,020**	**53,445**	**41,112**	**1,824**	**2,062**	**1,690**
**Infections averted[*] by prevention intervention scenario-** N (%)						
ART in FSWs (eART: 0.57)						
Increasing coverage to 25%	964 (4.0)	5,188 (9.7)	2,073 (5.0)	1 (0.04)	51 (2.5)	-16 (-1.0)
Increasing coverage to 50%	2,714 (11.3)	14,151 (26.5)	5,799 (14.1)	339 (18.6)	583 (28.3)	248 (14.7)
Increasing coverage to 81%	5,006 (20.8)	24,367 (45.6)	9,936 (24.2)	453 (24.8)	928 (45.0)	351 (20.8)
ART in FSWs (eART: 0.96)						
Increasing coverage to 25%	3,491 (14.5)	12,418 (23.2)	4,985 (12.1)	474 (26.0)	615 (29.8)	311 (18.4)
Increasing coverage to 50%	6,401 (26.6)	26,315 (49.2)	10,797 (26.3)	891 (48.8)	1,257 (61.0)	698 (41.3)
Increasing coverage to 81%	10,476 (43.6)	42,507 (79.5)	17,745 (43.2)	919 (50.4)	1,673 (81.1)	757 (44.8)
Condom use (eCondom: 0.80)						
Increasing use to 50%	NA	NA	NA	747 (40.9)	809 (39.2)	331 (19.6)
Increasing use to 80%	4,600 (19.2)	11,178 (20.9)	4,372 (10.6)	1,359 (74.5)	1,501 (72.8)	710 (42.0)
VMMC (eVMMC: 0.58)						
Increasing coverage to 50%	1,959 (8.2)	10,331 (19.3)	4,235 (10.3)	NA	NA	NA
Increasing coverage to 80%	4,422 (18.4)	21,626 (40.5)	8,904 (21.7)	NA	NA	NA
PrEP in FSWs (ePrEP: 0.51)						
Increasing coverage to 25%	2,796 (11.6)	3,038 (5.7)	1,042 (2.5)	294 (16.1)	172 (8.3)	54 (3.2)
Increasing coverage to 50%	5,715 (23.8)	6,238 (11.7)	2,134 (5.2)	557 (30.5)	290 (14.1)	118 (7.0)
Intervention packages						
Moderately optimistic scenario[†]	9,604 (40.0)	32,672 (61.1)	13,302 (32.4)	1,131 (62.0)	1,428 (69.2)	625 (37.0)
Most optimistic scenario[‡]	16,084 (67.0)	48,583 (90.9)	20,591 (50.1)	1,556 (85.3)	1,924 (93.3)	867 (51.3)
**Countries**	**Tunisia**			**Yemen**		
	**In FSWs**	**In clients**	**In client spouses**	**In FSWs**	**In clients**	**In client spouses**
**Cumulative incidence 2020-2030**	**210**	**261**	**189**	**257**	**302**	**265**
**Infections averted[*] by prevention intervention scenario-** N (%)						
ART in FSWs (eART: 0.57)						
Increasing coverage to 25%	NA	NA	NA	48 (18.7)	54 (17.8)	43 (16.2)
Increasing coverage to 50%	4 (2.0)	27 (10.3)	1 (0.5)	45 (17.6)	81 (26.7)	37 (14.0)
Increasing coverage to 81%	6 (2.9)	67 (25.8)	3 (1.6)	62 (24.1)	126 (41.8)	63 (23.8)
ART in FSWs (eART: 0.96)						
Increasing coverage to 25%	NA	NA	NA	69 (26.7)	82 (27.3)	55 (20.8)
Increasing coverage to 50%	77 (36.8)	120 (46.1)	40 (21.2)	85 (33.1)	151 (49.9)	76 (28.7)
Increasing coverage to 81%	106 (50.3)	205 (78.4)	77 (40.7)	128 (49.7)	240 (79.5)	110 (41.5)
Condom use (eCondom: 0.80)						
Increasing use to 50%	NA	NA	NA	68 (26.5)	77 (25.6)	49 (18.5)
Increasing use to 80%	98 (46.4)	119 (45.4)	37 (19.6)	178 (69.4)	208 (69.1)	120 (45.3)
VMMC (eVMMC: 0.58)						
Increasing coverage to 50%	NA	NA	NA	NA	NA	NA
Increasing coverage to 80%	NA	NA	NA	NA	NA	NA
PrEP in FSWs (ePrEP: 0.51)						
Increasing coverage to 25%	24 (11.2)	-2 (-0.8)	-14 (-7.4)	57 (22.0)	50 (16.6)	25 (9.4)
Increasing coverage to 50%	57 (27.0)	24 (9.0)	-3 (-1.6)	133 (51.8)	122 (40.4)	86 (32.5)
Intervention packages						
Moderately optimistic scenario[†]	92 (43.8)	129 (49.5)	44 (23.3)	139 (53.9)	180 (59.5)	92 (34.7)
Most optimistic scenario[‡]	164 (78.0)	232 (88.8)	91 (48.1)	214 (83.3)	276 (91.6)	140 (52.8)

Abbreviations: ART: antiretroviral therapy; FSWs: female sex workers; e: effectiveness; NA: not applicable; PrEP: pre-exposure prophylaxis; VMMC: voluntary medical male circumcision.

[*]Estimates for the number of averted infections have been rounded to the nearest digit and may not exactly match the corresponding proportion of averted infections.

[†]Includes expanding ART coverage to 50% with efficacy in preventing HIV transmission to partners of 96%, increasing condom use to 50%, and increasing PrEP to 25%. Baseline coverage was used whenever it was higher than that set in the investigated scenario. For South Sudan only, this package also included increasing VMMC to 50%.

[‡]Includes expanding interventions to the highest modelled coverage levels including expanding ART coverage to 81% with efficacy of 96%, increasing condom use to 80%, and increasing PrEP to 50%. For South Sudan only, this package also included increasing VMMC to 80%.

***Table S4:* Estimates of numbers and proportions of HIV infections averted over 10 years by increasing the coverage of select interventions among FSWs in MENA.** This table includes results for countries with significant injecting drug use among FSWs. Baseline coverage was used whenever it was higher than that set in the investigated scenario.

**Countries**	**Bahrain**			**Iran**		
	**In FSWs**	**In clients**	**In client spouses**	**In FSWs**	**In clients**	**In client spouses**
**Cumulative incidence 2020-2030**	**11**	**4**	**2**	**1,748**	**1,710**	**669**
**Infections averted[*] by prevention intervention scenario**- N (%)						
ART in FSWs & PWID partners (eART: 0.57)						
Increasing coverage to 25%	NA	NA	NA	19 (1.1)	36 (2.1)	1 (0.1)
Increasing coverage to 50%	<1 (4.5)	<1 (6.9)	<1 (4.4)	261 (14.9)	408 (23.9)	74 (11.1)
Increasing coverage to 81%	3 (25.4)	2 (39.3)	<1 (20.7)	556 (31.8)	838 (49.0)	180 (26.9)
ART in FSWs & PWID partners (eART: 0.96)						
Increasing coverage to 25%	NA	NA	NA	273 (15.6)	304 (17.8)	49 (7.3)
Increasing coverage to 50%	3 (29.3)	2 (42.8)	<1 (24.3)	664 (38.0)	879 (51.4)	181 (27.1)
Increasing coverage to 81%	7 (66.6)	3 (81.6)	1 (50.0)	989 (56.6)	1,403 (82.1)	287 (42.9)
Condom use (eCondom: 0.80)						
Increasing use to 50%	<1 (3.9)	<1 (7.4)	<1 (2.6)	NA	NA	NA
Increasing use to 80%	1 (9.8)	2 (47.9)	1 (27.8)	532 (30.5)	711 (41.6)	133 (19.9)
PrEP in FSWs (ePrEP: 0.51)						
Increasing coverage to 25%	1 (11.4)	<1 (6.7)	<1 (1.7)	206 (11.8)	48 (2.8)	-13 (-1.9)
Increasing coverage to 50%	3 (24.4)	<1 (11.1)	<1 (5.3)	496 (28.4)	258 (15.1)	35 (5.2)
Intervention packages						
Moderately optimistic scenario[†]	4 (38.1)	2 (46.7)	<1 (23.7)	820 (46.9)	946 (55.3)	201 (30.0)
Most optimistic scenario[‡]	9 (76.6)	4 (91.1)	1 (52.2)	1,368 (78.2)	1,545 (90.4)	325 (48.6)
**Countries**	**Libya**			**Pakistan**		
	**In FSWs**	**In clients**	**In client spouses**	**In FSWs**	**In clients**	**In client spouses**
**Cumulative incidence 2020-2030**	**295**	**340**	**115**	**3,162**	**3,055**	**1,183**
**Infections averted[*] by prevention intervention scenario**- N (%)						
ART in FSWs & PWID partners (eART: 0.57)						
Increasing coverage to 25%	NA	NA	NA	204 (6.5)	392 (12.8)	53 (4.5)
Increasing coverage to 50%	11 (3.6)	15 (4.5)	2 (1.7)	634 (20.1)	1,019 (33.4)	232 (19.6)
Increasing coverage to 81%	52 (17.6)	112 (32.9)	20 (17.4)	961 (30.4)	1,579 (51.7)	318 (26.9)
ART in FSWs & PWID partners (eART: 0.96)						
Increasing coverage to 25%	NA	NA	NA	492 (15.6)	792 (25.9)	160 (13.5)
Increasing coverage to 50%	100 (34.0)	140 (41.3)	23 (20.0)	1,066 (33.7)	1,609 (52.7)	303 (25.6)
Increasing coverage to 81%	172 (58.3)	268 (78.9)	49 (42.6)	1,820 (57.6)	2,538 (83.1)	568 (48.0)
Condom use (eCondom: 0.80)						
Increasing use to 50%	NA	NA	NA	NA	NA	NA
Increasing use to 80%	NA	NA	NA	1,257 (39.8)	1,541 (50.4)	326 (27.6)
PrEP in FSWs (ePrEP: 0.51)						
Increasing coverage to 25%	36 (12.1)	14 (4.1)	1 (0.9)	501 (15.8)	306 (10.0)	76 (6.4)
Increasing coverage to 50%	75 (25.3)	35 (10.2)	4 (3.5)	908 (28.7)	525 (17.2)	110 (9.3)
Intervention packages						
Moderately optimistic scenario[†]	128 (43.3)	153 (44.9)	26 (22.6)	1,457 (46.1)	1,804 (59.0)	393 (33.2)
Most optimistic scenario[‡]	202 (68.6)	274 (80.5)	51 (44.3)	2,514 (79.5)	2,820 (92.3)	628 (53.1)

Abbreviations: ART: antiretroviral therapy; FSWs: female sex workers; e: effectiveness; NA: not applicable; PrEP: pre-exposure prophylaxis; PWID: people who inject drugs.

[*]Estimates for the number of averted infections have been rounded to the nearest digit and may not exactly match the corresponding proportion of averted infections.

[†]Includes expanding ART coverage to 50% with efficacy in preventing HIV transmission to partners of 96%, increasing condom use to 50%, and increasing PrEP to 25%. Baseline coverage was used whenever it was higher than that set in the investigated scenario.

[‡]Includes expanding interventions to the highest modelled coverage levels including expanding, ART coverage to 81% with efficacy of 96%, increasing condom use to 80%, and increasing PrEP to 50%.

References

1. Chemaitelly H, Weiss HA, Calvert C, Harfouche M, Abu-Raddad LJ. HIV epidemiology among female sex workers and their clients in the Middle East and North Africa: systematic review, meta-analyses, and meta-regressions. *BMC Medicine* 2019; **17**(1).
2. Hamilton DT, Handcock MS, Morris M. Degree distributions in sexual networks: a framework for evaluating evidence. *Sex Transm Dis* 2008; **35**(1): 30-40.
3. Handcock MS, Jones JH. Likelihood-based inference for stochastic models of sexual network formation. *Theor Popul Biol* 2004; **65**(4): 413-22.
4. Cuadros DF, Crowley PH, Augustine B, Stewart SL, Garcia-Ramos G. Effect of variable transmission rate on the dynamics of HIV in sub-Saharan Africa. *BMC Infect Dis* 2011; **11**: 216.
5. Omori R, Abu-Raddad LJ. Sexual network drivers of HIV and herpes simplex virus type 2 transmission. *AIDS* 2017; **31**(12): 1721-32.
6. Omori R, Chemaitelly H, Abu-Raddad LJ. Dynamics of non-cohabiting sex partnering in sub-Saharan Africa: a modelling study with implications for HIV transmission. *Sexually transmitted infections* 2015; **91**(6): 451-7.
7. Garnett GP, Anderson RM. Factors controlling the spread of HIV in heterosexual communities in developing countries: patterns of mixing between different age and sexual activity classes. *Philos Trans R Soc Lond B Biol Sci* 1993; **342**(1300): 137-59.
8. Abu-Raddad L, Akala FA, Semini I, Riedner G, Wilson D, Tawil O. Characterizing the HIV/AIDS epidemic in the Middle East and North Africa : Time for strategic action. Washington DC: The World Bank Press; 2010.
9. Mumtaz GR, Kouyoumjian SP, Hilmi N, et al. The distribution of new HIV infections by mode of exposure in Morocco. *Sexually transmitted infections* 2013; **89 Suppl 3**: iii49-56.
10. Kouyoumjian SP, El Rhilani H, Latifi A, et al. Mapping of new HIV infections in Morocco and impact of select interventions. *International journal of infectious diseases : IJID : official publication of the International Society for Infectious Diseases* 2018; **68**: 4-12.
11. Mumtaz GR, Weiss HA, Thomas SL, et al. HIV among people who inject drugs in the Middle East and North Africa: systematic review and data synthesis. *PLoS medicine* 2014; **11**(6): e1001663.
12. Mahmud S, Mumtaz GR, Chemaitelly H, et al. The status of hepatitis C virus infection among people who inject drugs in the Middle East and North Africa. *Addiction* 2020; **115**(7): 1244-62.
13. Freeman MF, Tukey JW. Transformations Related to the Angular and the Square Root. 1950: 607-11.
14. Miller JJ. The Inverse of the Freeman – Tukey Double Arcsine Transformation. *The American Statistician* 1978; **32**(4): 138-.
15. Barendregt JJ, Doi SA, Lee YY, Norman RE, Vos T. Meta-analysis of prevalence. *Journal of epidemiology and community health* 2013; **67**(11): 974-8.
16. DerSimonian R, Laird N. Meta-analysis in clinical trials. *Controlled clinical trials* 1986; **7**(3): 177-88.
17. Borenstein M. Introduction to meta-analysis. Chichester, U.K.: John Wiley & Sons; 2009.
18. The Joint United Nations Programme on HIV/AIDS (UNAIDS). Country fact sheets. Available from: http://www.unaids.org/en/regionscountries/countries/. Accessed on August 26, 2021. 2021.
19. The United States Agency for International Development (USAID). The Demographic and Health Surveys Program. Available from: https://dhsprogram.com/data/available-datasets.cfm. Accessed on: August 26, 2021. 2021.
20. Lagarias JC, Reeds JA, Wright MH, Wright PE. Convergence properties of the Nelder--Mead simplex method in low dimensions. *SIAM Journal on optimization* 1998; **9**(1): 112-47.

21. Wawer MJ, Gray RH, Sewankambo NK, et al. Rates of HIV-1 transmission per coital act, by stage of HIV-1 infection, in Rakai, Uganda. *J Infect Dis* 2005; **191**(9): 1403-9.
22. Pinkerton SD. Probability of HIV transmission during acute infection in Rakai, Uganda. *AIDS and behavior* 2008; **12**(5): 677-84.
23. Hollingsworth TD, Anderson RM, Fraser C. HIV-1 transmission, by stage of infection. *J Infect Dis* 2008; **198**(5): 687-93.
24. Awad SF, Abu-Raddad LJ. Could there have been substantial declines in sexual risk behavior across sub-Saharan Africa in the mid-1990s? *Epidemics* 2014; **8**: 9-17.
25. Abu-Raddad LJ, Patnaik P, Kublin JG. Dual infection with HIV and malaria fuels the spread of both diseases in sub-Saharan Africa. *Science* 2006; **314**(5805): 1603-6.
26. Abu-Raddad LJ, Longini IM, Jr. No HIV stage is dominant in driving the HIV epidemic in sub-Saharan Africa. *AIDS* 2008; **22**(9): 1055-61.
27. Stover J, Brown T, Marston M. Updates to the Spectrum/Estimation and Projection Package (EPP) model to estimate HIV trends for adults and children. *Sexually transmitted infections* 2012; **88 Suppl 2**: i11-6.
28. UNAIDS. UNAIDS Reference Group on Estimates, Modelling and Projections. 2007.
29. The Joint United Nations Programme on HIV/AIDS (UNAIDS). AIDS epidemic update 2007. Available from: https://data.unaids.org/pub/epislides/2007/2007_epiupdate_en.pdf. Accessed on April 3, 2021. 2007.
30. The Joint United Nations Programme on HIV/AIDS (UNAIDS). UNAIDS report on the global AIDS epidemic. Available from: https://www.unaids.org/en/resources/documents/2010/20101123_globalreport. Accessed on April 3, 2021. 2010.
31. The Joint United Nations Programme on HIV/AIDS (UNAIDS). HIV estimates with uncertainty bounds 1990-Present. Available from: https://www.unaids.org/en/resources/documents/2021/HIV_estimates_with_uncertainty_bounds_1990-present. Accessed on September 5, 2021. 2021.
32. Ghys PD, Zaba B, Prins M. Survival and mortality of people infected with HIV in low and middle income countries: results from the extended ALPHA network. *AIDS* 2007; **21 Suppl 6**: S1-4.
33. Mumtaz GR, Awad SF, Feizzadeh A, Weiss HA, Abu-Raddad LJ. HIV incidence among people who inject drugs in the Middle East and North Africa: mathematical modelling analysis. *Journal of the International AIDS Society* 2018; **21**(3): e25102.
34. United Nations Population Division. World population prospects 2019. Available from: https://esa.un.org/unpd/wpp/. Accessed on January 25, 2021. 2019.
35. Ministère de la Santé au Maroc. Etude de prévalence des IST chez les femmes consultantes en SMI/PF al Wilaya de Rabat-Sale.
36. Feelemyer J, Des Jarlais D, Arasteh K, Uuskula A. Adherence to antiretroviral medications among persons who inject drugs in transitional, low and middle income countries: an international systematic review. *AIDS and behavior* 2015; **19**(4): 575-83.
37. Cohen MS, Chen YQ, McCauley M, et al. Antiretroviral Therapy for the Prevention of HIV-1 Transmission. *N Engl J Med* 2016; **375**(9): 830-9.
38. Mountain E, Mishra S, Vickerman P, Pickles M, Gilks C, Boily MC. Antiretroviral therapy uptake, attrition, adherence and outcomes among HIV-infected female sex workers: a systematic review and meta-analysis. *PLoS One* 2014; **9**(9): e105645.
39. Bhatta DN, Adhikari R, Karki S, Koirala AK, Wasti SP. Life expectancy and disparities in survival among HIV-infected people receiving antiretroviral therapy: an observational cohort study in Kathmandu, Nepal. *BMJ Glob Health* 2019; **4**(3): e001319.

40. Nakagawa F, May M, Phillips A. Life expectancy living with HIV: recent estimates and future implications. *Curr Opin Infect Dis* 2013; **26**(1): 17-25.
41. Teeraananchai S, Kerr SJ, Amin J, Ruxrungtham K, Law MG. Life expectancy of HIV-positive people after starting combination antiretroviral therapy: a meta-analysis. *HIV medicine* 2017; **18**(4): 256-66.
42. The World Bank. Antiretroviral therapy coverage. Available from: https://data.worldbank.org/indicator/SH.HIV.ARTC.ZS. Accessed on August 4, 2021. 2017.
43. Weller S, Davis K. Condom effectiveness in reducing heterosexual HIV transmission. *Cochrane Database Syst Rev* 2001; (3): CD003255.
44. Hughes JP, Baeten JM, Lingappa JR, et al. Determinants of per-coital-act HIV-1 infectivity among African HIV-1-serodiscordant couples. *J Infect Dis* 2012; **205**(3): 358-65.
45. Pinkerton SD, Abramson PR, Turk ME. Updated estimates of condom effectiveness. *J Assoc Nurses AIDS Care* 1998; **9**(6): 88-9.
46. Auvert B, Taljaard D, Lagarde E, Sobngwi-Tambekou J, Sitta R, Puren A. Randomized, controlled intervention trial of male circumcision for reduction of HIV infection risk: the ANRS 1265 Trial. *PLoS Med* 2005; **2**(11): e298.
47. Bailey RC, Moses S, Parker CB, et al. Male circumcision for HIV prevention in young men in Kisumu, Kenya: a randomised controlled trial. *The Lancet* 2007; **369**(9562): 643-56.
48. Gray RH, Kigozi G, Serwadda D, et al. Male circumcision for HIV prevention in men in Rakai, Uganda: a randomised trial. *The Lancet* 2007; **369**(9562): 657-66.
49. Weiss HA, Quigley MA, Hayes RJ. Male circumcision and risk of HIV infection in sub-Saharan Africa: a systematic review and meta-analysis. *AIDS* 2000; **14**(15): 2361-70.
50. Morris BJ, Wamai RG, Henebeng EB, et al. Estimation of country-specific and global prevalence of male circumcision. *Popul Health Metr* 2016; **14**: 4.
51. Fonner VA, Dalglish SL, Kennedy CE, et al. Effectiveness and safety of oral HIV preexposure prophylaxis for all populations. *AIDS* 2016; **30**(12): 1973-83.
52. Abu-Raddad LJ, Hilmi N, Mumtaz G, et al. Epidemiology of HIV infection in the Middle east and North Africa. *Aids* 2010; **24**(SUPPL. 2): S5-S23.
53. Al-Thani A, Abdul-Rahim H, Alabsi E, et al. Prevalence of Chlamydia trachomatis infection in the general population of women in Qatar. *Sexually transmitted infections* 2013; **89 Suppl 3**(Suppl 3): iii57-60.
54. Alrajhi AA, Halim MA, Al-Abdely HM. Mode of transmission of HIV-1 in Saudi Arabia. *Aids* 2004; **18**(10): 1478-80.
55. Ramezani A, Mohraz M, Gachkar L. Epidemiologic situation of human immunodeficiency virus (HIV/AIDS patients) in a private clinic in Tehran, Iran. *Archives of Iranian medicine* 2006; **9**(4): 315-8.
56. Omori R, Nagelkerke N, Abu-Raddad LJ. HIV and herpes simplex virus type 2 epidemiological synergy: misguided observational evidence? A modelling study. *Sexually transmitted infections* 2018; **94**(5): 372-6.
